# Bend and Snap: Computationally Exploring the Helix Bias for Classifying Abl Kinase Allosteric Modulators

**DOI:** 10.1021/acs.jcim.6c01990

**Published:** 2026-08-06

**Authors:** Pedro Tenório T. F. Leite, Philipe O. Fernandes, Diego M. Martins, Rafael Lopes Almeida, Ina Pöhner, Adolfo H. Moraes, Thales Kronenberger, Vinícius G. Maltarollo

**Affiliations:** † Departamento de Produtos Farmacêuticos, Faculdade de Farmácia, 28114 Universidade Federal de Minas Gerais (UFMG), Minas Gerais 31, Belo Horizonte 270-901, Brazil; ‡ Departamento de Química, Instituto de Ciências Exatas, Universidade Federal de Minas Gerais (UFMG), Minas Gerais 31, Belo Horizonte 270-901, Brazil; § School of Pharmacy, Faculty of Health Sciences, University of Eastern Finland, Kuopio 70211, Finland; ∥ Interfaculty Institute of Microbiology and Infection Medicine (IMIT), University of Tübingen, Tübingen 72076, Germany; ⊥ Partner-site Tübingen, German Center for Infection Research (DZIF), Tübingen 72076, Germany; # Laboratório de Ressonância Magnética de Alta Resolução (LAREMAR), Universidade Federal de Minas Gerais (UFMG), Belo Horizonte, Minas Gerais 31270-901, Brazil

## Abstract

Allosteric modulation of Abl kinase via its myristoyl binding pocket is a promising strategy for drug discovery, either aiming at inhibition to treat Chronic Myeloid Leukemia, or activation, under investigation for breast cancer therapy. Although activators and inhibitors can be differentiated by the induced α-helix-I conformation, *in silico* classification has been proven challenging. Our study distinguishes these classes effectively by integrating multiple computational approaches that account for the conformational plasticity of the regulatory α-helix-I. By evaluating traditional molecular docking, co-folding, and molecular dynamics simulations with methodology benchmarking, we use these methods to uncover mechanistic principles of allosteric modulation. Docking highlighted potentially stabilizing C–F interactions with deep-pocket residues, while co-folding correctly predicted helix bending for inhibitors and outperformed docking in virtual screening. Molecular dynamics revealed that the α-helix-I samples multiple possible conformations and uncovered motions consistent with dynamic coupling between helix bending and long-range restraint of the activation loop, a nuanced mechanism that static models could not elucidate. This study provides a validated framework that combines efficient classification with mechanistic analysis by combining molecular docking, co-folding, and molecular dynamics. Our approach aids in identifying myristoyl pocket ligands, differentiating inhibitors from activators, and suggesting allosteric principles that govern their function, paving the way for more rational design of next-generation, function-specific Abl modulators.

## Graphical abstract

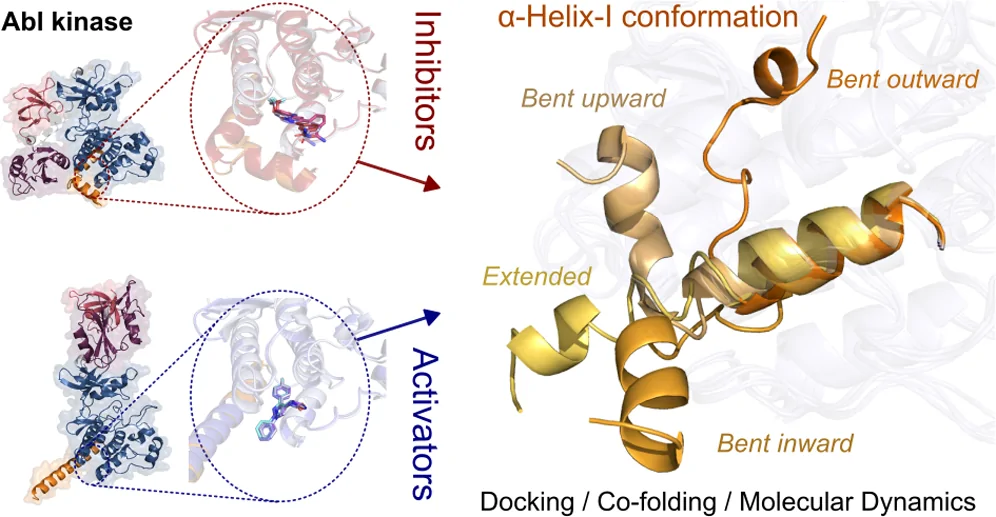

## 1 Introduction

Abelson (Abl) tyrosine kinase is a well-characterized enzyme, largely due to its clinical relevance in Chronic Myeloid Leukemia (CML). Abl is composed of a kinase domain, two Src homology (SH) domains acting as regulatory domains (SH2 and SH3), and a conserved disordered N-terminal portion. Abl has a complex self-regulatory mechanism coordinated, among other factors, by the interaction of its N-terminal myristoyl group and the myristoyl pocket (MP) in the kinase domain (Figure 1A,B).^1^ This interaction stabilizes the assembled state (mostly associated with inactivity) of Abl (Figure 1A). The disruption of this self-regulatory mechanism, as observed in the oncogene Bcr-Abl, is associated with CML. Bcr-Abl results from a fusion gene, located on the Philadelphia chromosome, which transcribes into an overactive enzyme due to the loss of the myristoylation site.^2^

**Figure 1:**
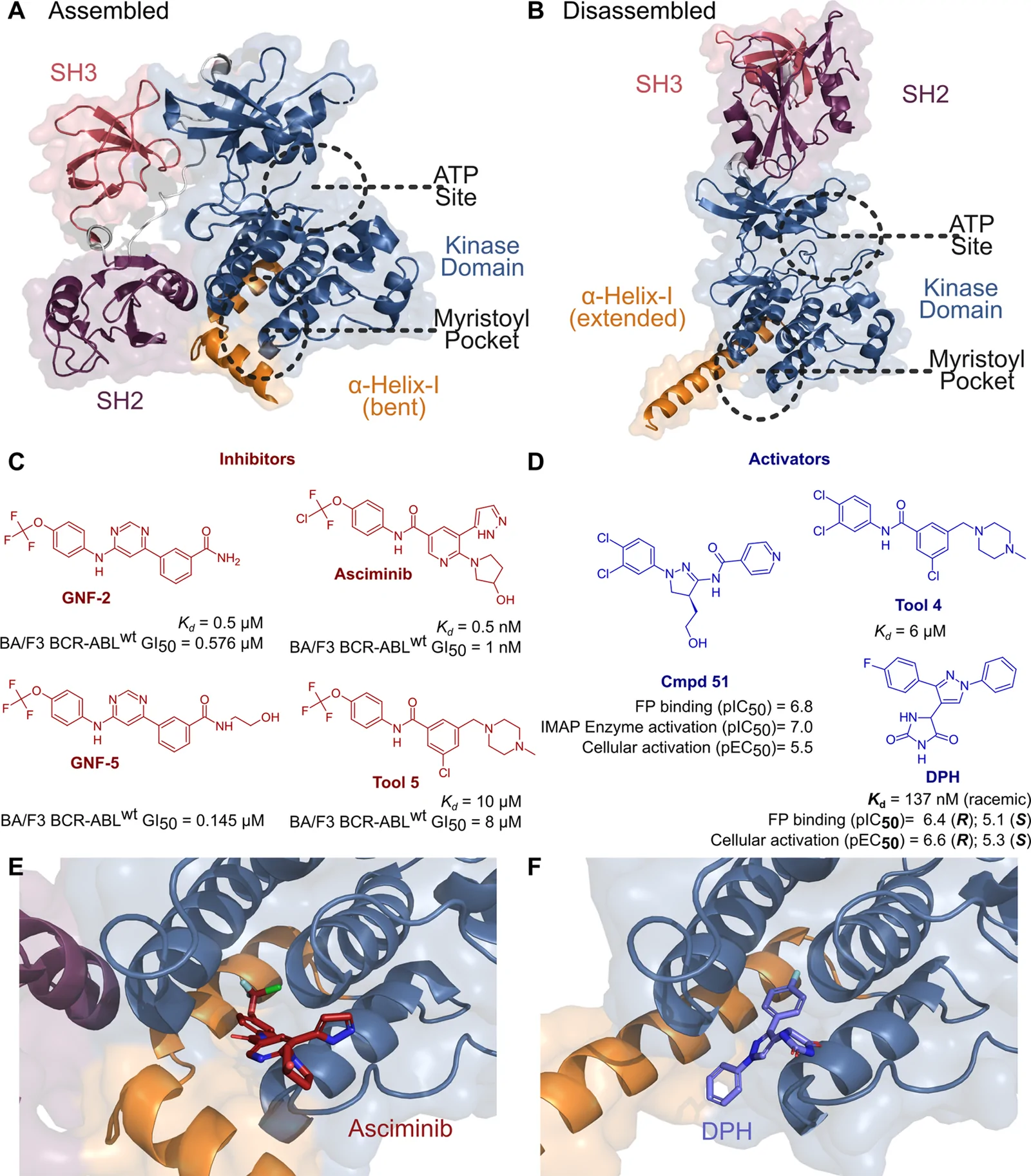
Assembled (A) crystal structure (PDB ID 5MO4) and Disassembled (B) model (modeled with AlphaFold2 using 1OPL as a template) of Abl kinase.^3,4^ Structures of allosteric inhibitors (C) and activators (D) with their respective dissociation constants and known activities.^4–8^ Close-up view of the myristoyl pocket with bound inhibitor (E) and activator (F).^4,8^

Despite the loss of myristoylation in Bcr-Abl, the MP is still conserved and enables allosteric inhibition by small molecules (Figure 1E,F).^9,10^ This is the mechanism of action of Asciminib, the latest FDA-approved drug for CML treatment, and the only FDA-approved allosteric Abl inhibitor to date.^6,11,12^ Allosteric modulation of kinase activity is particularly attractive because it acts on less conserved noncatalytic sites, potentially increasing selectivity and avoiding common resistance mechanisms.^11^ Their synergistic or additive effects with orthosteric inhibitors are also a relevant factor, which enables dual targeting of Bcr-Abl and combinatory therapy.^4,13,14^ Conversely, MP targeting also allows allosteric activation of Abl (Figure 1F)^7,8^ by stabilizing the disassembled conformation and competing with myristoyl. These small-molecule activators are under investigation due to the role of Abl activation in suppressing breast cancer cell growth and reducing mammary tumorigenesis.^15,16^

The main discerning structural feature for allosteric inhibitors and activators of Abl is the bending of its α-helix-I (Figure 1A,B). Inhibitors stabilize a specific inward-bent conformation, facilitating interaction between the kinase and regulatory domains and, consequently, stabilizing the inactive state.^17^ Modulator classes can be differentiated by analyzing helix bending of ligand-Abl complexes with NMR.^18^ Despite this structural difference in Abl conformation, distinguishing between activators and inhibitors remains a challenge for drug development. The tool compounds **Tool 4** and **Tool 5** (Figure 1C,D), discovered during the development of Asciminib,^6^ largely share a chemical core but differ in aromatic ring substituents. **Tool 4** was originally designed as an inhibitor to avoid steric clashes with the α-helix-I.^6^ However, **Tool 4** binds to the MP without stabilizing the inward-bent helix conformation, making the compound a potential competitive activator. The replacement of two chlorine atoms with the established pharmacophoric group –OCF_3_ in **Tool 5** recovered its ability to bend α-helix-I. Curiously, neither group directly interacts with the conformationally flexible region of α-helix-I, and therefore, the precise molecular mechanism underlying their impact on allosteric modulation remains unknown.

We evaluated how well computational techniques can distinguish allosteric Abl kinase inhibitors from activators and identified the most distinctive interactions, in addition to the established helix-bending. Traditional molecular docking, while valuable, is inherently limited by its reliance on static, predefined protein conformations. This limits the study of MP-binding ligands, whose function depends on α-helix-I-bending. Molecular dynamics (MD) simulations can model this flexibility,^19,20^ but at a high computational cost, which limits screening throughput.

To address these limitations, we explore co-folding as a complementary computational strategy. Specifically, we employ Boltz-2,^21^ a model that predicts the protein–ligand complex structure directly from the protein sequence and ligand SMILES without requiring a structural template. The model operates through a conditional reverse diffusion process that iteratively denoises randomly initialized atomic coordinates, progressively refining them into a physically plausible structural hypothesis from which binding affinity and probability are subsequently predicted. This approach allows the adaptation of the protein backbone, including the critical α-helix-I, to the chemical features of a bound ligand. Rather than simply benchmarking co-folding against docking, we integrated these methods to investigate how ligand-induced conformational changes differ between activators and inhibitors. Our combined approach seeks to evaluate co-folding’s efficacy not only in predicting accurate binding modes but also in distinguishing ligand-induced α-helix-I conformations. Additionally, we assessed the kinase domain’s conformational landscape in MD simulations and observed correlated motions. Our findings suggest an effective pipeline for virtual screening and a mechanistic framework for understanding allosteric modulators of Abl.

## 2 Methods

### 2.1 Analysis of Crystallographic Data for the Myristoyl Pocket

All crystal structures covering the kinase domain of human Abl (Uniprot ID P00519) and its close homologue, mouse Abl (Uniprot ID P00520), were obtained from RCSB PDB^22^ (Supporting Information, Table S1). DFG and αC-helix conformations were classified based on KLIFS records^23^ when available, and by visual inspection for all other cases. All chains of the available structures were split and aligned using PyMOL Open Source v3.2.0a0. A myristoyl consensus pocket was derived from all residues located within 5 Å of any small-molecule binder of the MP. A pairwise sequence alignment of the canonical FASTA sequences was generated with EMBOSS Needle^24^ with default parameters. The consensus pocket residues were visualized to inspect MP pocket residue conservation (Figure S1A).

To assess conformational variability of the MP captured in the crystallographic data, a heavy-atom RMSD was computed for each MP residue. In addition, each residue of each structure in the structural ensemble (Table S1) was individually aligned based on its backbone atoms and C_β_ to orient the side chain, and per-residue root-mean-square fluctuations (RMSF) of the MP residue coordinates were calculated (Figure S2).

### 2.2 Protein Structure Selection and Modeling

Two crystal structures of Abl kinase were selected as representative rigid receptors for docking studies since they had the highest resolution for the two classes of allosteric modulators, captured the conformational trends in the crystallographic data well (Figure S2), and had a fully resolved α-helix-I: PDB 3K5V (inhibitor-bound, bent α-helix-I, mouse Abl)^25^ and 3PYY (activator-bound, extended α-helix-I, human Abl)^8^ (Supporting Information, Table S1). For those, we generated two human Abl working models (UniProt P00519, residues 242–510) as input for the MD simulations with Maestro (version 2024.3). The first, representing the bent α-helix-I, was based on the mouse Abl PDB 3K5V and generated using the homology modeling workflow in Maestro with default settings. For the extended α-helix-I (based on human Abl PDB 3PYY), missing portions of the activation loop were fixed with Prime.^26^ The MP of human and mouse Abl is composed of virtually identical residues, with the Asn336 in the human enzyme being replaced by serine in mouse Abl (see sequence alignment in Supporting Information, Figure S1A). Both residues face away from the MP toward bulk solvent (Figure S1B) and their side chains are not in contact with any MP ligands.

### 2.3 Molecular Docking Validation

Molecular docking was initially performed with AutoDock Vina and Glide on the prepared models. Prior to docking, cocrystallized ligands and water molecules were removed. Polar hydrogens were added, and Gasteiger partial charges were assigned using OpenBabel (v3.1.1).^27^ Ligand 3D structures were prepared with OpenBabel from ligand SMILES (see extended data set enclosed as Supporting Information) to assign protonation states, convert ligands to SDF (and later PDBQT format for AutoDock Vina). Energy minimization was performed using the MMFF94 force field.^28^

For AutoDock Vina (v1.2.0),^29^ different parameter combinations (box size and exhaustiveness) were systematically evaluated for pose prediction accuracy. Cubic boxes of sizes 20, 25, and 30 Å^3^ were centered on the MP using the center of mass of its established residues,^30^ and increasing exhaustiveness of 8, 16, and 32 was employed. Glide (v7.7)^31,32^ was used with its default settings, with standard precision.

The Glide grid was centered on the centroid of the cocrystallized ligands, with box inner box sizes adjusted to 10 Å^3^ and outer box sizes of 25 Å^3^.

To test whether pocket rigidity might bias the docking results, we allowed side-chain flexibility in AutoDock Vina and ADFR (v1.0)^33^ for the four most conformationally variable residues which had been observed as key contacts in rigid-receptor docking (Leu341, Ile432, Cys464, Val468; selected from rigid docking results and crystallographic RMSF, Supporting Information Figure S2), and, for a broader full pocket flexibility, including the residues described by Fallacara *et al.* (2014) and Martins *et al.* (2024) (Arg332, Leu340, Leu341, Leu429, Ile432, Glu462, Cys464, Val468, Phe493, Ile502, Val506, Leu510) with AutoDock Vina.^12,30^

ADFR docking used the same grid sizes and centroids as described above for Vina docking and was run with default parameters. Ligand SDF conversion to PDBQT utilized meeko (v0.6.1).^34^

For rigid docking, pose prediction capacity was assessed by redocking and cross-docking, specifically docking activators **DPH** and **Cmpd51** ^7,8^ into the 3PYY structure and inhibitors **GNF-2** and **Asciminib** ^4,25^ into the 3K5V structure. Pose accuracy was evaluated by computing the Root Mean Square Deviation (RMSD) of the best-scoring (AutoDock Vina’s score and Glide’s Docking score) poses.

### 2.4 Co-Folding Validation

Co-folding was performed with Boltz-2.^21^ Inputs were prepared in the YAML format using the human Abl sequence (UniProt P00519, residues 242–510, kinase domain), and SMILES for the ligands. During pose-prediction validation, initial tests revealed that in the absence of an orthosteric-site ligand MP ligands were misdirected to the ATP-binding site (Supporting Information, Figure S3). Therefore, imatinib and/or contact constraints were included in subsequent protocols, since it is present in the orthosteric binding pocket of ten out of 16 Abl structures with small molecules in the MP (Table S1). Each ligand–receptor complex was predicted using five protocols able to direct ligands to the MP: two template-based (**P1** and **P2**, using 3K5V and 3PYY, which were used in the docking protocol) and three template-free. Among the template-free protocols, one included distance constraints and no imatinib (**P3**), one had imatinib and no contact constraints (**P4**), and the last protocol included both (**P5**) (Supporting Information, Figure S4). The final template-free protocol included a distance constraint of 6.0 Å to residue Ala344 (i.e., the deep end of the MP), which enhanced pose accuracy (Supporting Information, Figure S4). Imatinib was similarly constrained to the ATP-binding site by Met318. Force flags were set to false to allow flexible adaptation. Pose accuracy was evaluated using RMSD, as in docking, with additional checks for correct helix bending.

### 2.5 Virtual Screening Validation

To evaluate each method’s ability to discriminate true MP ligands from inactive compounds, we performed virtual screening (VS) validation with libraries of 52 activators and 280 inhibitors with activities retrieved from Simpson *et al.* (2010) and Furet *et al.* (2016), respectively.^7,35^ Their predicted affinities were compared with those of 2,449 activator decoys (Decoy_A) and 4,650 inhibitor decoys (Decoy_I) generated by the DUD-E server,^36^ to ensure physicochemical similarity and topological dissimilarity. VS performance was evaluated with the Receiver Operating Characteristic Area Under the Curve (ROC AUC), and the Boltzmann-Enhanced Discrimination of Receiver Operating Characteristic (BEDROC, α = 20), which emphasizes early enrichment.^37^ The exponent prefactor α was set to 20 because it emphasizes enrichment in the top fraction of screened compounds while still capturing meaningful differences between protocols. Higher α values would complicate a comparison, while lower values would place less weight on early recognition and diminish the metric’s practical relevance for virtual screening. Molecular docking and receptor/ligand preparation followed the same protocols used for pose prediction described in 2.3. For both AutoDock Vina (rigid and flexible) and Glide, two docking strategies were compared: a single-structure approach, in which activators were docked to the 3PYY model and inhibitors to the 3K5V model, and an ensemble approach, in which each compound was docked against both receptor conformations and the best docking score was selected for ranking.

For the flexible docking approach with ADFR, due to the higher computational cost, activators and corresponding decoys were evaluated using the 3PYY model, and inhibitors and corresponding decoys using the 3K5V model.

Due to the higher computational cost of Boltz-2 compared to docking, only the best protocol (**P5**) was used for VS validation (see results and discussion), and each of its available output metrics (binding probabilities and predicted affinities) was evaluated for its ability to discriminate true binders from decoys. Similarly, for the following interaction fingerprints and feature analysis, AutoDock Vina was selected over Glide due to its superior performance in inhibitor VS validation.

### 2.6 Interaction Fingerprints and Feature Analysis

To move beyond affinity scores and identify specific interaction determinants for class differentiation, we analyzed interaction patterns for inhibitors and activators. Interaction Fingerprints (IFPs) were extracted using Arpeggio^38^ for all ligand–receptor complexes generated in VS validation. Separated matrices were generated with custom Python scripts for each method (AutoDock Vina and Boltz-2—available upon request), containing binary interaction fingerprints consisting of structural, residue, atom-pair, and interaction-type information, in addition to each software’s metrics and the α-helix-I angle for Boltz-2.

To reduce noise and identify the most discriminatory features, a Weighted Fisher Weight (wFW)^39^ analysis was implemented. Fisher Weight (FW) is a filter feature selection metric to quantify a feature’s ability to separate a class from one or more other classes.^40^ For a given feature, it was calculated as the ratio of between-class variance to within-class variance (eq 1)­

1 $${FW}_{AvsB}={\left(\mu_{A}-\mu_{B}\right)}^{2}/\left(\sigma_{A}^{2}-\sigma_{B}^{2}\right)$$

where μ and σ represent the mean and standard deviation, respectively, of the feature values for classes A and B.

A high FW indicates a feature whose values are both consistently different between two classes and relatively stable within each class. In our multiclass scenario (Activators, Inhibitors, Decoy_A, Decoy_I), a single binary comparison is insufficient. Therefore, we employed a wFW score (eq 2) that integrates five binary comparisons, with coefficients chosen to reflect the relative importance of each discrimination task, following a strategy similar to that of Araujo *et al.* (2015).^39^ The highest coefficient (0.30) was assigned to distinguish activators from inhibitors. Intermediate coefficients (0.20 each) were given to separate each active class from the combined pool of all compounds, and lower coefficients (0.15 each) were allocated to distinguish each active class from its corresponding decoys.

2 $$wFW=0.30{FW}_{Activators\;vs\;Inhibitors}+0.20{FW}_{Activators\;vs\;\text{all}}+0.20{FW}_{Inhibitors\;vs\;\text{all}}+0.15{FW}_{Activators\;vs\;Decoy\text{\_}A}+0.15{FW}_{Inhibitors\;vs\;Decoy\text{\_}I}$$

Features were then filtered by their wFW scores and interfeature correlations to reduce redundancy (Figure 2).

**Figure 2:**
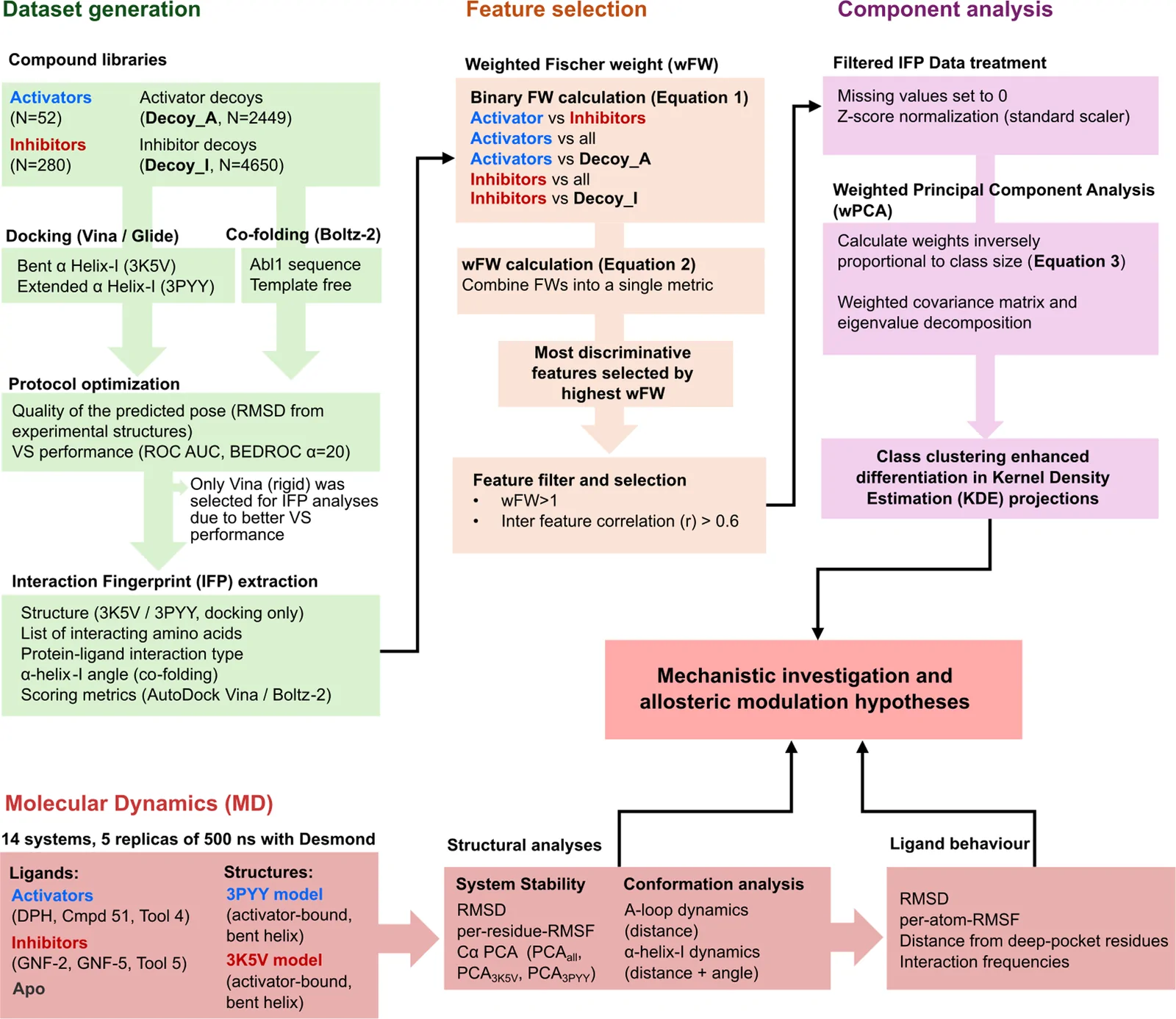
Flowchart describing the main steps in performing the wPCA for class clustering, combined with MD for mechanistic interpretations. IFP: interaction fingerprint; KDE: kernel density estimation; FW: fisher weight; PCA: principal component analysis; MD: molecular dynamics; RMSD: root-mean square deviation; RMSF: Root-mean square fluctuation.

Weighted Principal Component Analysis (wPCA) was performed on the filtered feature sets, scaled with SciKit-Learn’s Standard Scale (1.8.0),^41^ to verify class clustering and differentiation. Class weights (*w*_c_) were applied, inversely proportional to sample size to prevent decoy over-representation in the principal components (eq 3).

3 $$w_{c}=N/\left(C^{*}n_{c}\right)$$

where *N* is the total number of samples, *C* is the number of distinct classes, and *n*_c_ is the number of samples in class c.

This weighting scheme ensures that the total influence of each class on the covariance matrix is equal regardless of class size, balancing the contribution of all classes to the derived principal components and enabling a visualization that more accurately reflects separation based on feature variance.

### 2.7 Molecular Dynamics Simulations

We used the Desmond MD simulation engine^42^ and the OPLS4 force-field.^43^ Ligand charges and parameters for OPLS4 were generated during system preparation using the force-field builder tool (Maestro2024.3). The prepared systems were solvated in a cubic box, with a minimum distance of 13 Å between any protein atom and the box edges. The TIP3P water model^44^ was used to describe the solvent, and the net charge was neutralized using Na^+^ ions. RESPA integrator timesteps of 2 fs for bonded and near and 6 fs for far interactions were applied. The short-range Coulombic interactions were treated using a cutoff value of 9.0 Å, whereas long-range Coulombic interactions were estimated using the Smooth Particle Mesh Ewald (PME) method.^45^ Prior to production simulations, systems were relaxed using the default Desmond relaxation protocol. Briefly, Maestro′s Desmond implementation uses a default protocol that proceeds through two stages of energy minimization (backbone restrained and unrestrained) followed by four stages of MD runs with gradually diminishing restraints, which together constitute an automated multistage equilibration process. It minimizes the solute atoms under restraints while relaxing the solvent molecules and ions around the solute. Next, it minimizes the full system without restraints to remove residual strain. With the *NVT* ensemble (constant volume and temperature), a short MD simulation runs at low temperature (10 K) with solute restraints. This helps to gradually heat the solvent without disrupting the solute structure. It continues gradual heating to the target temperature (310 K) under *NPT* (constant pressure, constant temperature) ensemble, while maintaining positional restraints. This step allows solvent density to adjust to controlled conditions. Full relaxation under NPT conditions, without restraints, follows. For production, simulations were run in *NPT* ensemble at a temperature of 310 K (using the Nosé-Hoover thermostat^46,47^ ) and pressure of 1.01325 bar (Martyna-Tobias-Klein barostat^48^). Fourteen different systems were simulated (six ligands + apostructure for each model), each with imatinib bound to the orthosteric site. Starting structures modeled from the activator-bound (extended α-helix-I, PDB 3PYY) and the inhibitor-bound (bent α-helix-I, PDB 3K5V) conformations were used. Simulated systems included apo controls, the activators **DPH**, **Cmpd51**, and **Tool 4**, and inhibitors **GNF-2**, **GNF-5**, and **Tool 5**. Initial protein–ligand complexes were either predicted by Vina docking or, when available, derived from crystals by merging ligand coordinates into our models following structural alignment. Each system was simulated in five independent replicas of 500 ns, each with a random seed.

### 2.8 Structural Analyses of Molecular Dynamics and Co-Folded Structures

#### 2.8.1 Ligand Movement and System Stability

To assess ligand placement within the MP, the distance between the ligand center of mass and the center of mass of deep-pocket residues Ala344, Leu429, Val468, and Phe493 was monitored. Both protein and ligand RMSD and RMSF were computed with MDAnalysis (v2.7.0)^49,50^ to monitor system stability (Supporting Information, Figures S6–S8), after aligning each trajectory to its first frame by protein Cα atoms. Ligand interaction frequencies in MD simulations were also computed by exporting and processing each frame separately with Arpeggio and by using the Simulation Interaction Diagram tool (implemented in Maestro 2024.3).

#### 2.8.2 Principal Component Analysis

Cα Principal component analysis (PCA) was employed to characterize dominant backbone motions after aligning trajectories to a common reference (the first frame of the apo 3K5V trajectory) with MDAnalysis. Three PCA analyses were conducted: PCA_all_, with all 14 trajectories combined; PCA_3PYY_, with the seven 3PYY trajectories; and PCA_3K5 V_, with the seven 3K5V trajectories. Extreme motions of the combined trajectories were visualized using the modevectors Pymol (v3.0) script,^51^ considering motions with a distance greater than 4 Å between the lowest and highest projections for each evaluated principal component.

#### 2.8.3 α-Helix-I Conformation

The conformation of the α-helix-I was quantified by measuring the bending angle between Cα atoms of residues Ala487, Thr495, and Lys508, along with the distance between Cα atoms of helix residue Lys508 and C-lobe residue Lys467. These metrics were calculated for every frame of the MD trajectories and each static co-folded structure obtained from the VS validation.

#### 2.8.4 Activation Loop Movement

Sonti *et al.* (2018) have monitored the activation loop (A-loop) state with the distance between residues Lys356 (C-lobe) and Gly405 (A-loop).^52^ The same metric was used to evaluate the conformations adopted in both the MD trajectories and the co-folded structures, comparing ligand classes, similarly to the helix analyses.

## 3 Results and Discussion

### 3.1 Co-Folding Accurately Predicts Binding Modes and Outperforms Docking in Virtual Screening Metrics

We first evaluated whether co-folding could reproduce experimental binding mode of MP ligands. Initial tests showed that MP ligands were misdirected to the ATP-binding site in the absence of an orthosteric-site ligand or without using contact constraints (Supporting Information Figure S3). This behavior has been reported previously for allosteric ligands and attributed to the overrepresentation of orthosteric complexes in the PDB.^53^ Moreover, the binding probabilities predicted by Boltz-2 were systematically higher when the ligand was correctly directed to the MP (Supporting Information Figure S3). We therefore incorporated imatinib in the ATP site and applied a distance constraint (protocol **P5**), which placed all reference ligands in the MP with heavy-atom RMSD values comparable to rigid docking. Furthermore, the protocol additionally correctly predicted the class-specific α-helix-I conformation (extended for activators, Figure 3A; bent inward for inhibitors, Figures 3C and S3). Notably, all reported crystallographic structures of MP ligands also contain a ligand in the ATP-binding site (Supporting Information Table S1). These results indicate that co-folding achieves optimal pose accuracy and correct helix bending under conditions that closely resemble the available experimental structures with which co-folding models were trained.

**Figure 3:**
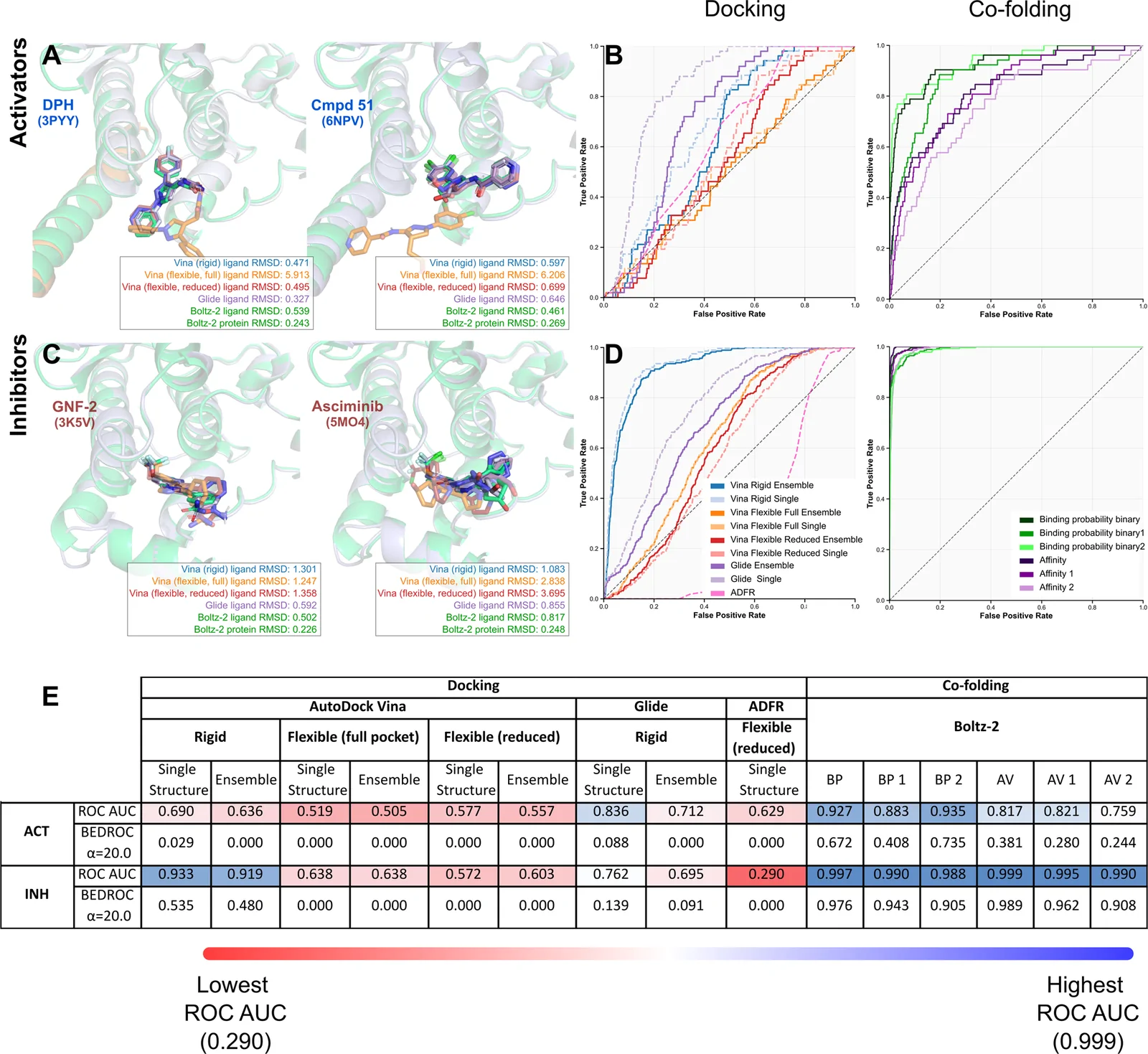
Comparison of docking and co-folding for Abl myristoyl pocket ligands. (A) Pose-prediction accuracy for the activators **DPH** and **Cmpd 51**, measured by heavy-atom RMSD of the top-ranked pose relative to the crystallographic reference (3PYY and 6NPV, respectively). Methods compared: rigid-receptor docking with AutoDock Vina (Vina rigid) and Glide; flexible side-chain docking with AutoDock Vina using a full-pocket set (Vina flexible full) or a reduced set (Vina flexible reduced); and co-folding with Boltz-2 (protocol P5). For Boltz-2, the protein Cα RMSD relative to the 3PYY template is also shown. (B) Virtual-screening performance for activators presented as ROC curves. (C) Corresponding pose-prediction accuracy for the inhibitors **GNF-2** and Asciminib, referenced to 3K5V and 5MO4, respectively. For Boltz-2 protein RMSD, the 3K5V template was used. (D) Virtual-screening performance for inhibitors presented as ROC curves. (E) Area under the ROC curve (AUC) and Boltzmann-enhanced discrimination of ROC (BEDROC, α = 20) values for all methods.

Flexible-side-chain docking with AutoDock Vina degraded pose accuracy relative to the rigid protocol (Figure 3A,C), demonstrating that the pocket’s limited plasticity does not benefit from explicit sampling in the current docking framework. This poor performance carried over to virtual screening, where rigid docking also outperformed the flexible protocols, and single-structure docking yielded better class discrimination than ensemble docking.

Co-folding outperformed docking programs in virtual screening considering both AUC and BEDROC enrichment (α = 20) using Binding Probability (BP) for activators (Figure 3B) and all metrics (BP and Affinity Value [AV]) for inhibitors (Figure 3D). A recent rigorous assessment of Boltz-2 applied to ultra-large virtual screening data sets^54^ reported a notable difference between BP and AV. While the BP classifier demonstrated strong performance in distinguishing true binders from nonbinders, the AV metric proved insensitive to ligand pose quality and binding-site mutations, and showed poor correlation with experimental affinity values (Figure 3E).

Our implementation differs methodologically from the cited “out-of-the-box” application, which relied solely on target sequences and compound SMILES as inputs. The inclusion of contact restraints and an additional orthosteric ligand allowed us to tailor the protocol to achieve accurate ligand pose and proper folding of α-helix-I. Given the reported limitations of the AV metric for ranking actives, we evaluated the performance of both BP and AV within a classification framework to establish a robust and specific protocol for Abl kinase MP ligands.

The optimal Boltz-2 metric proved to be class-dependent: BP values were best for activators (AUC = 0.935 with BP2), whereas AV achieved near-perfect (AUC = 0.999) discrimination for inhibitors. This divergence suggests the metrics leverage distinct aspects from the training. The AV regressor, trained on different available measurement types (*K*_*i*_, *K*_*d*_, IC_50_, EC_50_) might be particularly attuned to local chemical variation and structure–activity relationships, rendering this metric sensitive to the chemical signatures characteristic of inhibition. Conversely, the probability classifier may capture broader patterns of target engagement learned from diverse binary binding data, including synthetic decoys.^21^

This empirical class-dependence aligns with the developers’ recommendation to use BP for hit discovery and AV for lead optimization, and highlights that both metrics can be valuable when selecting according to ligand class.

These results position co-folding as a powerful methodology for VS against the Abl MP pocket. Despite its higher computational cost relative to conventional molecular docking, co-folding yielded score metrics more closely linked to functional class in our case study. Due to the superior performances of co-folding and rigid docking, they were selected for further IFP analyses.

### 3.2 Interaction Fingerprints as a Tool to Distinguish Activators, Inhibitors, and Decoys

To elucidate the specific interactions underlying class discrimination among activators, inhibitors, and decoys, we analyzed Interaction Fingerprints (IFPs). wFW analyses suggest clear differences in the most discriminative features derived from docking and co-folding approaches (Figure 4A,B). In particular, Boltz-2 IFP highlighted a shallow-pocket C–C hydrophobic interaction with residue Cys464 as a key discriminator, whereas deep-pocket interactions such as C–F contacts involving Leu341, Ala344, and Ile432 were identified from docking simulations (Figure 4C–E). Our analysis of the existing crystallographic data on MP modulators highlighted Ile432 and Cys464 as distinct between our two template structures and indicated a greater variation in Cys464 conformations within the extended α-helix-I class structures compared to the bent class (Figure S2). We thus attribute the observed difference to the fact that the co-folding protocol, by default, generates a single ligand-adapted protein conformation per complex, including the class-specific α-helix-I bending and residue orientations, whereas docking attempts to fit ligands to two static helix conformations and pocket residue orientations. Importantly, the point mutations Ala344Leu and Cys464Tyr are known to confer resistance to allosteric inhibitors,^55^ likely due to steric clashes with the larger side chains of leucine and tyrosine, reinforcing the functional relevance of Ala344 and Cys464 and highlighting the complementarity of analyzing the results of both modeling methods.

**Figure 4:**
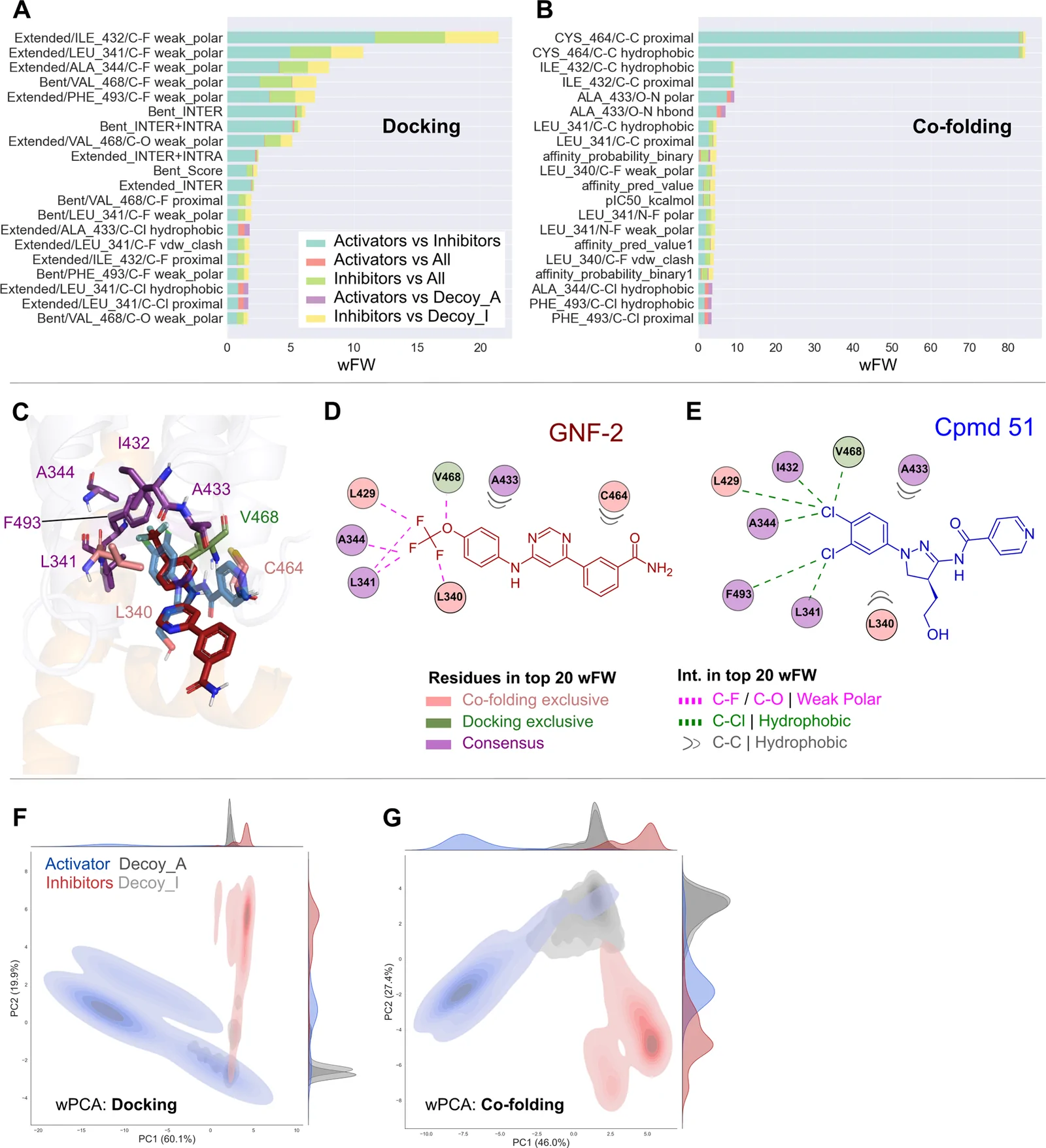
Top 20 feature contributions to the weighted Fisher Weight (wFW, eq 2) score for (A) docking and (B) co-folding results. The *Y* axis shows the top features, while the *X* axis shows their respective wFW. The colored bars indicate the contribution of each binary FW after applying eq 3 to obtain wFW. (C) Structural mapping of residues corresponding to the top features: co-folding-exclusive (salmon), docking-exclusive (green), and consensus (purple), overlaying co-folded structures of **GNF-2** and **Cmpd 51**. Exclusive features are present among the top 20 features ranked by wFW. Diagrams showing interactions in the top 20 wFW for **GNF-2** (D) and **Cmpd 51** (E). Weighted Principal Component Analysis (wPCA) for the (F) docking and (G) co-folding data sets, after wFW and correlation filters.

The deep-pocket amino acid Ile432 appears as a consensus interaction across both methods: co-folding favored a C–C hydrophobic interaction, whereas docking predominantly identified weak polar C–F contacts, which may again partly reflect the differing residue orientations in the static receptor structures. Docking results align with previously reported structure–activity relationship (SAR) studies,^5,6^ which established the CF_3_ group (or CF_2_Cl, in the case of Asciminib) as important for inhibitory activity. These observations suggest that while our implemented Boltz-2 protocol excels at structure prediction and ligand classification, multiple-conformation docking still provides complementary insights into dynamics-dependent interactions, and a combined workflow offers the most comprehensive mechanistic view.

Subsequently, wPCA improved clustering of inhibitors on the Boltz-2 data set, whereas activators showed some overlap with decoys in both wPCAs (Figure 4F,G). In both analyses, the first principal component (PC1) effectively separated activators from inhibitors, while the second principal component (PC2) distinguished actives from decoys (with better discrimination for inhibitors), confirming that the derived features capture functionally relevant variance.

Examination of the PC loadings reveals the molecular features contributing to these separations (Supporting Information, Figure S5). In the docking-based wPCA, PC1 is dominated by C–Cl interactions involving the extended helix conformation, with residues Leu341, Ala433, and Val468. For co-folding, the loadings are more evenly distributed across multiple interaction types and pocket regions. PC1 separates activators from inhibitors primarily through carbonyl interactions (Glu462), proximal contacts (Leu341, Ala433, Ile502), and hydrophobic interactions (Leu341, Pro465, Val468). Boltz-2’s affinity prediction value and binding affinity probability heavily influence PC2, differentiating both active classes from decoys, as seen in the VS performance. C–F weak polar interactions (Leu340) contribute significantly to both PC1 and PC2, reinforcing the critical role of this pharmacophoric feature identified in our wFW analysis. This is also consistent with the findings of Schoepfer *et al.* (2018), who describe a favorable polar interaction between one of the fluorine atoms of inhibitors with the carbon atom of the backbone of Leu340.^6^ Notably, because correlation filtering following wFW removes highly correlated features, chemically related interactions, such as the cluster of C–F interactions with deep-pocket residues, associated with the inhibitor CF_3_ motif, are represented in the wPCAs by a subset of these residues (Leu341 for docking wPCA and Leu340 in co-folding wPCA) rather than all of them.

### 3.3 Structural Analyses in Molecular Dynamics and Co-Folded Structures

The molecular dynamics simulations described below sample conformational space in 500 ns over five replicas and reveal correlations between ligand class, α-helix-I bending, and activation-loop dynamics. Throughout this section, we interpret observed correlated motions and conformational preferences as structural hypotheses that are consistent with available experimental data and that provide a plausible mechanistic framework for allosteric modulation. Direct testing of causal links (*e.g.,* by mutagenesis or kinetic measurements) lies beyond the scope of this study.

#### 3.3.1 3K5V Model Shows Higher Stability for all Holo and Apo Systems

The trajectories starting from 3K5V models (bent helix) had greater stability than trajectories starting from 3PYY models (extended helix). Residue RMSF values show that the A-loop and the α-helix-I are the most fluctuating regions in the MD simulations, and both have lesser movement in 3K5V-starting trajectories (Figure 5A). The same trend is observed in Cα PCA, where a broad landscape is observed for 3PYY starting trajectories (Figures 5B and S10–S12), while 3K5V trajectories have more localized distribution. These smaller conformational changes in 3K5V likely arise from starting in a stable global minimum, thereby limiting the exploration of alternative states within the employed simulation time. In contrast, the diverse conformational landscape observed for 3PYY appears to stem from an unfavorable starting point, which allows ligand interaction to more visibly steer the system’s dynamic trajectory.

**Figure 5:**
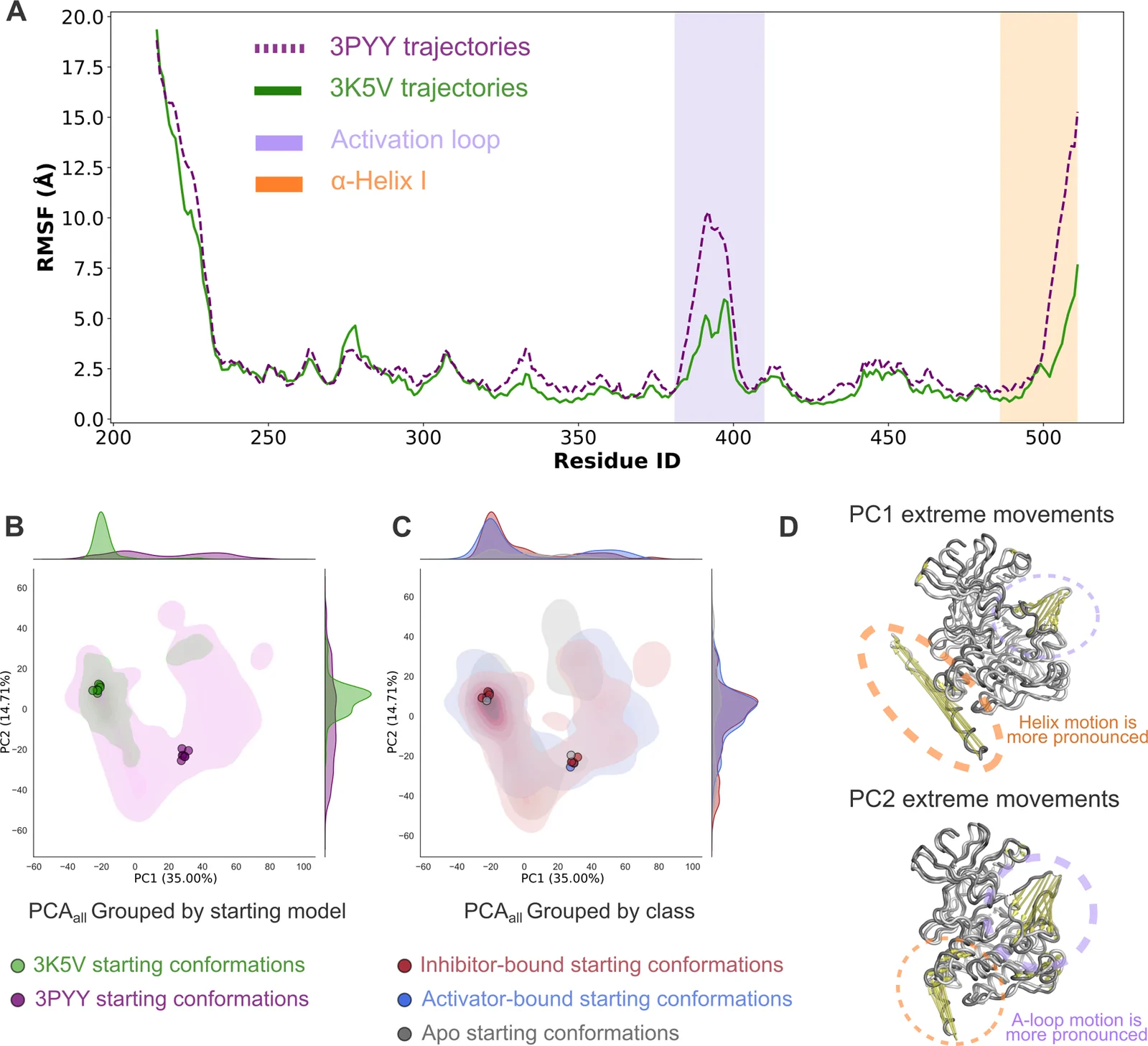
Comparative stability of MD simulations initiated from the inhibitor-bound (3K5V, bent α-helix-I) and activator-bound (3PYY, extended α-helix-I) models. (A) Mean Cα RMSF as a function of residue number, averaged over all 3K5V-started (solid line) and all 3PYY-started (dashed line) trajectories. The activation loop (A-loop) and α-helix-I regions are highlighted. (B) Conformational landscapes from principal component analysis (PCA) of all concatenated trajectories, projected onto the first two principal components (PC1 and PC2). Colored contours represent kernel density estimates for 3K5V-started (green) and 3PYY-started (purple) systems. (C) Corresponding PC1-PC2 landscapes for holo (red for inhibitors, blue for activators) and apo (gray) trajectories. (D) Extreme-motion projections along PC1 and PC2, visualized with modevectors. Yellow arrows indicate the direction and relative amplitude of backbone displacement between the minimum and maximum projections. PC1 is dominated by α-helix-I bending, while PC2 primarily captures A-loop rearrangement. The thickness of dashed circles indicates the impact of each region in PC1 and PC2.

Helix movement played a more prominent role in PC1 and the A-loop in PC2, as observed in extreme motion projections (Figure 5D). Inhibitors appear to access an Abl conformational subspace that is not accessed by activators (Figures 5C and S10C–I). This observation indicates that inhibitor-bound and activator-bound trajectories differently occupy the conformational landscape, which is defined by the coordinated motion involving both the A-loop and α-helix-I residues (Figure 5D). Although we cannot affirm that the presented MD simulations establish causality, class-specific effects on regulatory mechanisms beyond the local helix conformation were observed throughout the trajectories. Specific A-loop and α-helix-I conformations are further discussed in the following sections, and ligand stability within the MP is discussed in the Supporting Information (section Ligand Dynamics Within the MP Suggests Distinct Deep-Pocket Occupance in MD Trajectories).

#### 3.3.2 Molecular Dynamics Simulations Suggest Multiple Conformations in α-Helix-I

The α-helix-I conformation is the established experimental determinant of the modulator class.^56^ In MD simulations, four distinct conformations were identified, namely: bent upward, extended, bent outward, and bent inward (Figure 6A–D). The outward-bent conformation is observed only in one trajectory (Figure 6K). The low frequency observed for the fully extended helix (similar to PDB 3PYY) across all trajectories, even though half of them start in this conformation, suggests it might not be prominent in a dynamic system. This is further evidenced by its presence in crystal structures being described in the literature as the result of crystal packing.^8^ The bent inward (inhibitor-bound) conformation is more prevalent even in the activator MD simulations, highlighting the difficulty of discerning class from MD snapshots alone and the complexity of the conformational landscape. This indicates the relationship between α-helix-I and A-loop might be relevant to accessing the inhibitor-exclusive conformation observed in the backbone PCA.

**Figure 6:**
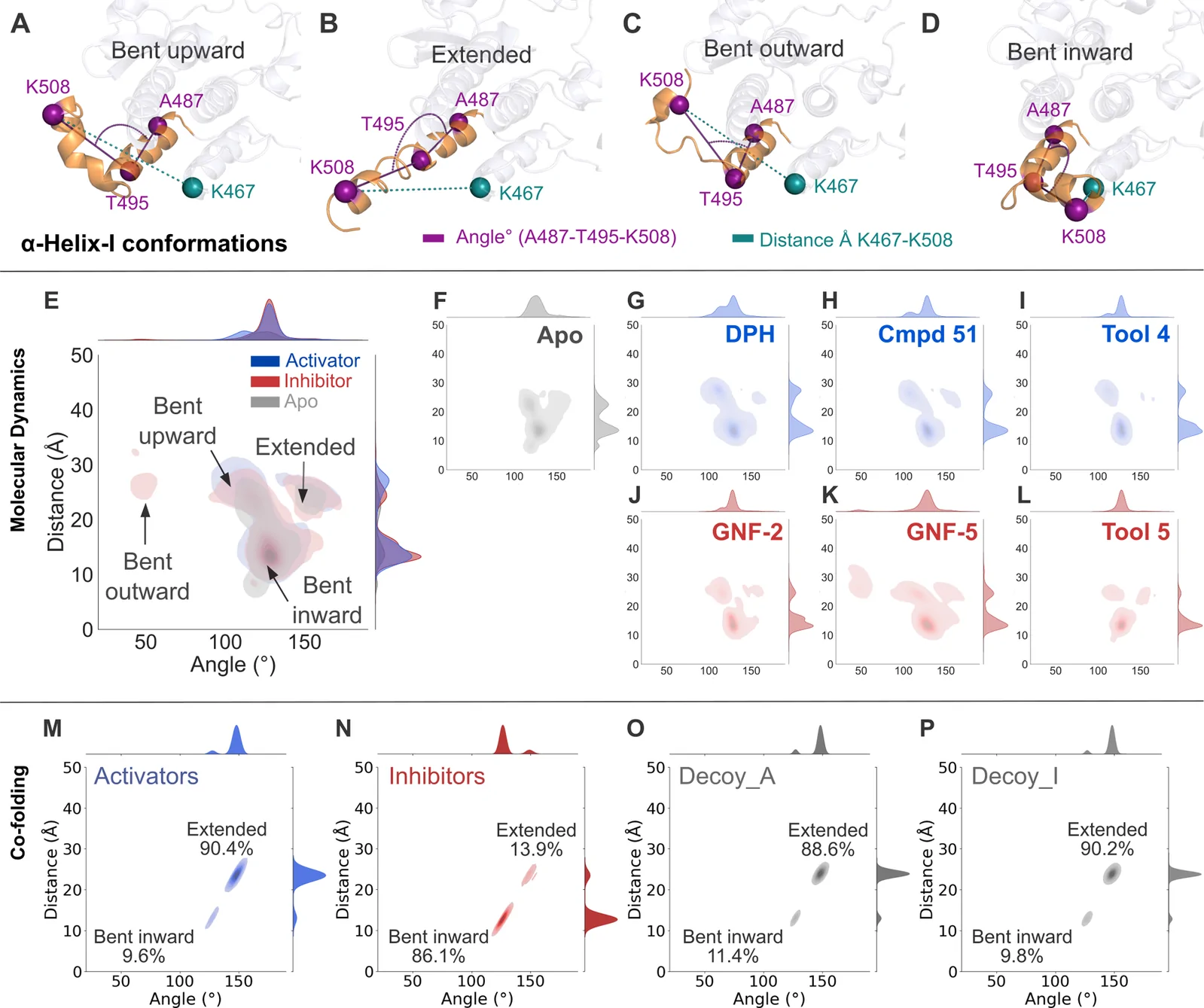
Four major conformations adopted by the α-helix-I in the trajectories: (A) bent upward, (B) extended, (C) bent outward, (D) bent inward. Kernel density estimation (KDE) plots showing these conformations, with distance and angle measures for the combined trajectories (E) and the individual apo (F), activator (G-I), and inhibitor (J–L) trajectories. KDE plots showing helix conformation for Boltz-2 predicted structures of the activators (M), inhibitors (N), activator decoys (O), and inhibitor decoys (P) data sets. Simulation results were obtained from the merged trajectory of 5 replicas, each 500 ns long.

Increasing computational and experimental evidence positions this coupling as a central axis of allosteric modulation in Abl kinase. Using hydrogen exchange mass spectrometry, Iacob *et al.* (2011) demonstrated that binding of the allosteric inhibitor **GNF-5** to the MP induces conformational changes not only to the α-helix-I, but also to the ATP binding site.^57^ This effect was preserved in the imatinib-resistant Thr315Ile mutant, which also enabled the orthosteric inhibitor dasatinib to bind to Abl Thr315Ile, an interaction that was not observed in the absence of **GNF-5**.

Conversely, Zhang *et al.* (2022) investigated the interaction between both sites using MD and community network analysis, showing that the Thr315Ile mutation disrupts correlated motions between the MP and the ATP site, and that this disruption could be overcome with the combination of allosteric and orthosteric inhibitors.^58^

Beyond this direct coupling between sites, a related body of work has examined how ATP-site ligands can drive large-scale conformational rearrangements of the Abl regulatory core. Using NMR, Skora and colleagues demonstrated that imatinib stabilizes the disassembled Abl core, and that the assembled state is reestablished in the presence of **GNF-5**.^59^ The authors attribute the imatinib-induced conformational shift to its binding to the ATP site with nanomolar affinity, which induces a rotation of the N-lobe that disrupts interactions with the SH3 domain.^52,59,60^ Conversely, Xie *et al.* (2022) reported that imatinib can also bind directly to the MP with micromolar affinity, stabilizing an extended α-helix-I conformation, which the authors propose as an alternative explanation for the disassembled state observed in the Abl-imatinib complex.^61^ Although these studies propose different primary drivers of core disassembly upon imatinib binding, they collectively establish that ligand interaction with either the ATP site or the MP can trigger long-range conformational changes in Abl. Our MD trajectories reflect the structural changes associated with this network organization within the kinase domain, suggesting possible correlated motions that agree with the experimentally observed long-range communication. The inhibitor-associated conformational subspace we observe is characterized by the coordinated motion of α-helix-I and the A-loop (Figure 5D), suggesting a dynamically correlated state that may be related to allosteric communication between both structural elements.

Clearly in contrast, co-folded structures predicted only two states (bent inward and extended), with a strong tendency: inhibitors predominantly adopted the bent helical conformation, whereas activators and decoys favored the extended conformation (Figure 6M–P). This evidence shows that the co-folding protocol can effectively discriminate between modulator classes when analyzing helix conformation, providing a simpler, clearer distinction between activators and inhibitors compared with MD simulations. Its bias toward generating extended helices rather than other possible conformations (such as the bent-upward state observed in MD) in noninhibitor complexes likely reflects the composition of the crystallographic training data, where extended and bent-inward conformations are the only states represented, compared with the more diverse conformational ensemble accessed by MD.

Simulations comparing the isolated Abl kinase domain with versions featuring the Abl-regulatory domains^19^ show that only the Myr-free kinase domain displays such displacement of the α-helix-I. Indeed, Myr-bound simulations of both isolated kinase domains and kinase + regulatory domains primarily show the inward-bent conformation. Despite the relevant time scale (2 μs), those simulations were generated in singlicate, which makes their confident interpretation difficult.

Previous larger simulation studies starting from the Abl active conformation (ATP-bound) also did not observe α-helix-I straightening in the absence of myristic acid. In contrast, simulations starting from an extended α-helix-I unfolded regardless of the presence of myristoyl in the pocket.^20^ The bent inward α-helix-I conformation was shown to remain folded in myristoyl absence but also to be strongly destabilized in their simulated time scale (high RMSF values, 20 × 2 μs).^20^ Buhr *et al.* have quantified that longer extended helices can be stabilized by another protein copy packed against the first in the crystal lattice and suggested that this conformation might be a crystallization artifact.

#### 3.3.3 Inhibitors Restrain Distal Activation Loop Dynamics

Monitoring the Lys356-Gly390 distance (C-lobe and A-loop, respectively, Figure 7C) revealed that inhibitors consistently hindered transitions between A-loop states from the less stable starting position in the extended trajectories, whereas activators showed broader distributions, akin to the apo simulations (Figure 7A), except for **DPH**. This indicates that inhibitors might not only stabilize helix bending, but also enforce a restrained conformation on the distal activation loop, potentially stabilizing an intermediate allosteric state incompatible with activation. This would help explain why activators without the clear steric clash between **DPH** and the helix are unable to inhibit Abl, revealing a complex, multifactorial dynamic relationship between the modulator and the enzyme. Similarly to the helix analysis, co-folded structures showed a clear distinction between inhibitors and other ligands via the A-loop distance distribution (Figure 7B).

**Figure 7:**
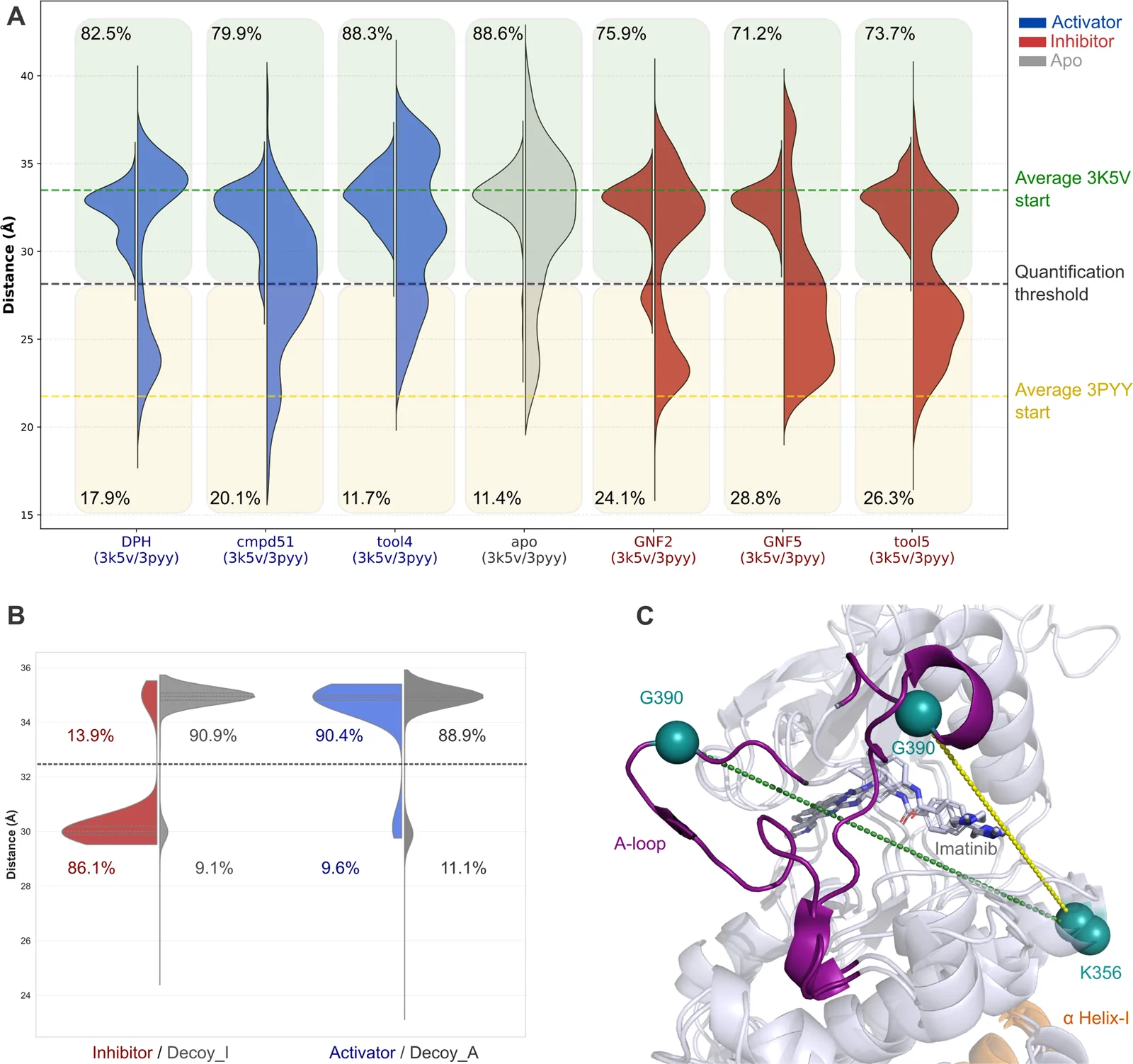
(A) A-loop to C-lobe distance distribution for each trajectory. Threshold for quantification (27.61 Å) was determined as the mean between the average starting distances of 3K5V model trajectories (33.48 Å) and 3PYY model trajectories (21.75 Å). Quantification for each ligand was computed for both 3K5V and 3PYY model trajectories. (B) A-loop to C-lobe distance distribution for Boltz-2 predicted structures of each ligand class. (C) Illustration of different MD trajectory points, showing the Lys356-Gly390 distance above (dashed green line) and below (dashed yellow line) the quantification threshold for MD trajectories. Simulation results were obtained from the merged trajectory of 5 replicas, each 500 ns long.

## 4 Conclusions

Our study established a protocol to effectively distinguish allosteric activators from inhibitors of Abl kinase using multiple approaches that account for MP conformational plasticity, particularly of α-helix-I. We compared binding pose accuracy and virtual screening performance and also achieved deeper mechanistic insight by using traditional molecular docking, co-folding, and MD simulations.

Our co-folding protocol performed VS and functionally classified MP ligands better than docking methods. While achieving pose accuracy comparable to docking, it surpassed the docking-based discrimination between true modulators and decoys: AUC increased from 0.836 (best docking protocol) to 0.935 (best co-folding metric) for activators, and from 0.933 to 0.999 for inhibitors. Similarly, BEDROC enrichment at α = 20 increased from 0.088 to 0.735 for activators and from 0.535 to 0.989 for inhibitors. Our co-folding protocol performed VS and functionally classified MP ligands better than docking methods. While achieving pose accuracy comparable to docking, it surpassed the docking-based discrimination between true modulators and decoys: AUC increased from 0.836 (best docking protocol) to 0.935 (best co-folding metric) for activators, and from 0.933 to 0.999 for inhibitors. Similarly, BEDROC enrichment at α = 20 increased from 0.088 to 0.735 for activators and from 0.535 to 0.989 for inhibitors.

A key advantage of co-folding is the direct prediction of the α-helix-I conformation, which is critical for modulator function. Unlike the heterogeneous ensemble sampled by MD, co-folding, after protocol optimization, converged on a clear binary distinction via helix bending. Inhibitors predominantly stabilized the bent-inward conformation, whereas activators favored the extended state. This ability to provide a structure-based prediction of functional class from a single prediction run represents a significant advance for *in silico* VS campaigns targeting this system. Our co-folding protocol, however, required optimization, including the use of an orthosteric ligand and distance constraints, to correctly direct ligands to the MP and recover the functional helix conformation. This tailoring reflects the biases inherent in training data and the particular structural features of the Abl myristoyl pocket. Extending the approach to other allosteric systems will likely require analogous system-specific adjustments, guided by available structural knowledge. The MD-based observations of coupled motions are consistent with experimental data, and further causal validation with mutagenesis or kinetic experiments is a perspective for future work.

Additionally, a multimethod perspective remains relevant for mechanistic understanding. Our work shows that the strengths of traditional and next-generation methods are complementary for this system. While co-folding excelled at classification, docking-based interaction fingerprinting successfully identified complementary deep-pocket pharmacophoric interactions that align with established SARs and mutation studies (notably, IFPs obtained from docking highlight Ala344, whereas IFPs obtained from co-folding highlight Cys464). Furthermore, MD uncovered multiple possible helix-bending conformations and revealed correlated motions between α-helix-I bending and long-range restraint of A-loop that were not observable in static models. These observations suggest a potential coupling mechanism that warrants further experimental investigation.

This study demonstrates co-folding as a potential method for addressing the long-standing challenge in Abl allosteric drug discovery, achieving classification performance acceptable according to the literature standard after parameter optimization. However, we believe that a combination of approaches could benefit a more complete understanding of the studied system (in this case, MP ligands, considering class discrimination and virtual screening performance with co-folding and physically reliable interaction profile with docking) to this date. Our approach aids in identifying MP ligands and differentiating inhibitors from activators, and suggests allosteric principles that govern their function, paving the way for more rational design of next-generation, function-specific Abl modulators.

## Supplementary Material





## Data Availability

Prepared structures, MD trajectories, MD simulation configuration and parameter files, as well as raw and processed data for Abl-ligand interactions, are available in the Zenodo repository (under the DOIs: 10.5281/zenodo.18375176). Crystallographic structures used are available from https://www.rcsb.org/. Third-party software employed in the manuscript were as follows. ChemBioDraw Ultra version 18.0 (https://perkinelmerinformatics.com/) is distributed under license. Autodock Vina version 1.2 (https://vina.scripps.edu/) and AutoDockFR (ADFR, https://ccsb.scripps.edu/adfr/) areopen-source free software. Maestro versions 2024v3 (https://newsite.schrodinger.com/platform/products/maestro/), and Glide version 7.7 (https://newsite.schrodinger.com/platform/products/glide/) are distributed under the Schrödinger’s license. OMEGA version 3.1.1.2 (https://www.eyesopen.com/omega) and QUACPAC version 2.0.1.2 (https://www.eyesopen.com/quacpac) are distributed under the OpenEye Scientific license.Third-party software employed in the manuscript include: GraphPad PRISM version 10.2 (https://www.graphpad.com/), Schrödinger Suite 2024.3 (https://www.schrodinger.com) and PyMOL version 2.5.2 (https://pymol.org/) are distributed under respective licenses.

## Notes

Smiles for all used ligands are available in an extended data set (XLSX)
Supporting figures, data collection, and further information are available. All molecular dynamics trajectories and raw data related to the protein–ligand interactions within the simulations will be made available in the Zenodo repository upon publication, with the following (PDF)

## Abbreviations

EC_50_: half-maximal effective concentration
LN: ligand efficiency
MD: molecular dynamics simulations
PCA: principal component analysis
RMSD: root-mean-square deviation
SD: standard deviation
CML: chronic myeloid leukemia
MP: myristoyl pocket
NMR: ML, Machine learning
VS: virtual screening
ROC AUC: ROC Area under the curve, BEDROC
IFP: interaction fingerprint
FW: Fischer weight
wFW: weighted Fischer weight.
