# Supplementary material for: Bend and Snap: Computationally Exploring the Helix Bias for Classifying Abl Kinase Allosteric Modulators
Source: J Chem Inf Model. 2026 Aug 6;66(16):10302–17. doi: 10.1021/acs.jcim.6c01990 (PMC13509006; doi:10.1021/acs.jcim.6c01990)
Supplement: Supplementary file 1 [file ci6c01990_si_002.pdf]

**Supporting Information**

# Bend and snap: computationally exploring the helix bias for classifying Abl kinase allosteric modulators

Pedro Tenório T. F. Leite,[1*] Philipe O. Fernandes,[1*] Diego M. Martins,[2] Rafael Lopes Almeida,[3] Ina Pöhner,[3] Adolfo H. Moraes,[2,5] Thales Kronenberger,[3,4#] Vinícius G. Maltarollo[1#]

1 Departamento de Produtos Farmacêuticos, Faculdade de Farmácia, Universidade Federal de Minas Gerais (UFMG), Belo Horizonte, Minas Gerais 31.270-901, Brazil.

2 Departamento de Química, Instituto de Ciências Exatas, Universidade Federal de Minas Gerais (UFMG), Belo Horizonte, Minas Gerais 31.270-901, Brazil;

3 School of Pharmacy, Faculty of Health Sciences, University of Eastern Finland, 70211, Kuopio, Finland.

4 *a* Interfaculty Institute of Microbiology and Infection Medicine (IMIT), University of Tübingen, Tübingen, Germany. *b* Partner-site Tübingen, German Center for Infection Research (DZIF), Tübingen, Germany.

5 Laboratório de Ressonância Magnética de Alta Resolução (LAREMAR), Universidade Federal de Minas Gerais (UFMG), 31270-901, Belo Horizonte, MG, Brazil.

* These authors equally contributed to this work.

[#] Corresponding authors: V.G.M, viniciusmaltarollo@gmail.com, Departamento de Produtos Farmacêuticos, Faculdade de Farmácia, Universidade Federal de Minas Gerais (UFMG), Belo Horizonte, Minas Gerais 31.270-901, Brazil. T.K., thales.kronenberger@uni-tuebingen.de Partner-site Tübingen, German Center for Infection Research (DZIF), DE-72076, Tübingen, Germany.

**Table S1.** Summary of Abl crystallographic data. If α-helix-I configurations are labeled as extended in the below table, residues after the kinked region of the bent helix (from Ser501, see **Figure S1**) had to be present, otherwise the helix was labeled as cut. Light gray background: *Mus musculus* Abl, white background: human Abl. Italics: mutation in MP. All compounds were labeled by their PDB ligand identifiers with names of MP modulators discussed in the manuscript additionally reported in parentheses. STI = imatinib.

| PDB-ID | Resolution [Å] | DFG | aC-helix | Orthosteric ligand | Myristoyl pocket ligand | α-helix-I configuration |
|---|---|---|---|---|---|---|
| 3k5v | 1.74 | out | in | STI | STJ (GNF-2) | bent |
| 5mo4 | 2.17 | out | in | NIL | AY7 (asciminib) | bent |
| 3pyy | 1.85 | out | in | STI | 3YY (DPH) | cut [A], extended [B] |
| 6npv | 1.86 | out | In | STI | KWP (Cmpd-51) | cut [A], extended [B] |
| 1opl | 3.42 | out-like | in | P16 | MYR (myristic acid) [A], - [B] | bent [A], cut [B] |
| 2fo0 | 2.27 | out-like | in | P16 | MYR (myristic acid) | bent |
| 8ssn | 2.86 | in | out | SKI | AY7 (asciminib) | bent |
| 4xey | 2.89 | out-like | in | 1N1 | - | extended |
| *2f4j (H396P)* | 1.91 | in | in | VX6 | - | extended |
| 2v7a | 2.50 | in | in | 627 | - | extended |
| 2hyy | 2.40 | out | in | STI | - | cut |
| 2hz4 | 2.80 | in | in | 4ST [B], - [A, C] | - | cut |
| 2hz0 | 2.10 | out | in | GIN | - | cut |
| 2e2b | 2.20 | out | in | 406 | - | extended |
| *2g2h (H396P)* | 2.00 | out-like | in | P16 | - | extended |
| 3ue4 | 2.42 | out-like | in | DB8 | - | extended |
| 4yc8 | 2.90 | out-like | in | 4B7 | - | cut |
| 6npe | 2.15 | out | in | STI | KWD | cut [A], extended [B] |

| | | | | | | |
|---|---|---|---|---|---|---|
| 6npu | 2.33 | out | in | STI | KWV | cut [A], extended [B] |
| 4zog | 2.30 | out-like | in | VX6 | - | cut [A], extended [B] |
| 7cc2 | 2.72 | out-like | in | FVC | - | cut |
| 7dt2 | 2.30 | out-like | in | HJ9 | - | cut |
| 7w7y | 2.20 | out-like | in | 8IW | - | cut |
| 2gqg | 2.40 | in | in | 1N1 | - | cut |
| 2hzi | 1.70 | out-like | in | JIN | - | cut |
| 3cs9 | 2.21 | out | in | NIL | - | cut |
| 5hu9 | 1.53 | out | in | 66K (2nd allosteric) | (EDO) | cut |
| 7w7x | 2.00 | out-like | in | 8DW | - | cut |
| 8h7f | 2.45 | out-like | in | QEW | - | cut |
| 8h7h | 2.28 | out-like | in | QH9 | - | cut |
| 8i7s | 1.95 | in | in | 6CI | - | cut |
| 8i7t | 2.80 | in | in | 6I5 | - | cut |
| 8i7z | 2.25 | in | in | 6CI | - | cut |
| 9ks5 | 2.20 | in | in | - | - | cut |
| 3qri | 2.10 | out | in | 919 | (NA) [A], - [B] | cut |
| 3qrj | 1.82 | out | in | 919 | - | cut |
| 3qrk | 2.30 | out | in | 9DP | - | cut |
| 7n9g | 2.20 | out-like | in | 1N1 | STI (imatinib) | cut |
| 2hiw | 2.20 | out | in | 7MP | - | cut |
| *6bl8 (N336S)* | 2.50 | out-like | in | PVB | - | extended |
| 4twp | 2.40 | in | in | AXI | - | extended |
| 4wa9 | 2.20 | out-like | na/in | AXI | - | extended |
| 1opk | 1.80 | out-like | in | P16 | MYR (myristic acid) | bent |
| 1fpu | 2.40 | out | in | PRC | - | cut |
| 1iep | 2.10 | out | in | STI | - | cut |
| 1m52 | 2.60 | out-like | in | P17 | - | extended |

| | | | | | | |
|---|---|---|---|---|---|---|
| 1opj | 1.75 | out | in | STI | MYR (myristic acid) | bent |
| 2hzn | 2.70 | out | in | KIN | - | cut |
| 2qoh | 1.95 | in | in | P3Y | - | cut [A], extended [B] |
| 2z60 | 1.95 | in | in | P3Y | - | extended |
| 3kf4 | 1.90 | in | in | B90 | - | extended [A], cut [B] |
| 3kfa | 1.22 | out | in | B91 | - | extended |
| 3ms9 | 1.80 | out | in | STI | MS9 | cut |
| 3mss | 1.95 | out | in | STI | MS7 | cut |
| 6hd4 | 2.03 | out | in | STI | FYW | bent |
| 6hd6 | 2.30 | out | in | STI | FYH [A], - [B] | bent |
| 3ik3 | 1.90 | out | in | 0LI | - | extended |
| 3oxz | 2.20 | out | in | 0LI | - | extended |
| 3oy3 | 1.95 | out | in | XY3 | - | extended |
| 3dk3 | 2.02 | out-like | in | SX7 | - | extended, bent loop |
| 3dk6 | 2.02 | out-like | in | SX7 | - | extended, bent loop |
| 3dk7 | 2.01 | out-like | in | SX7 | - | extended, bent loop |

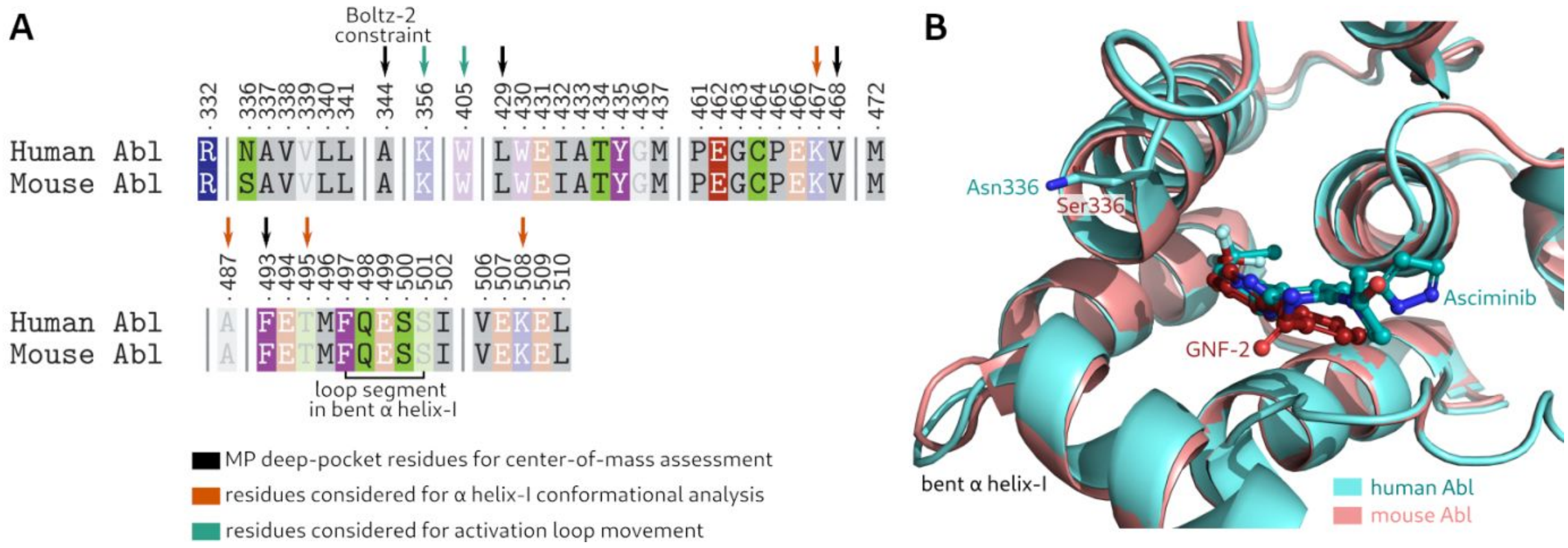


**Figure S1.** Sequence and structure comparison for human and mouse Abl myristoyl pockets. Pocket residues were defined by the consensus of residues within 5 Å of myristoyl pocket ligands in either protein (see **Table S1**). (A) Sequence alignment of human and mouse Abl (Uniprot IDs P00519 and P00520, respectively) with numbering corresponding to the human Abl canonical FASTA sequence. The residues chosen for the Boltz-2 distance constraint and deep-pocket center-of-mass definitions, and residues considered for the α-helix-I and activation loop conformational analysis are indicated by arrows. Residues are shaded based on type (red: acidic, blue: basic, purple: aromatic, green: polar, gray: apolar). Tinted regions correspond to residues not identified as part of the MP but connecting two sequence stretches or being discussed in the text. (B) Structural overlay of human Abl (PDB-ID 5MO4, cyan) and mouse Abl (3K5V, salmon) with ligands **Asciminib** and **GNF-2**, respectively (ball-and-stick). The only sequence difference, residues Asn336 and Ser336 in human and mouse Abl, respectively, are labeled. Residue side chains both point away from the pocket towards the solvent (or regulatory domains when assembled).

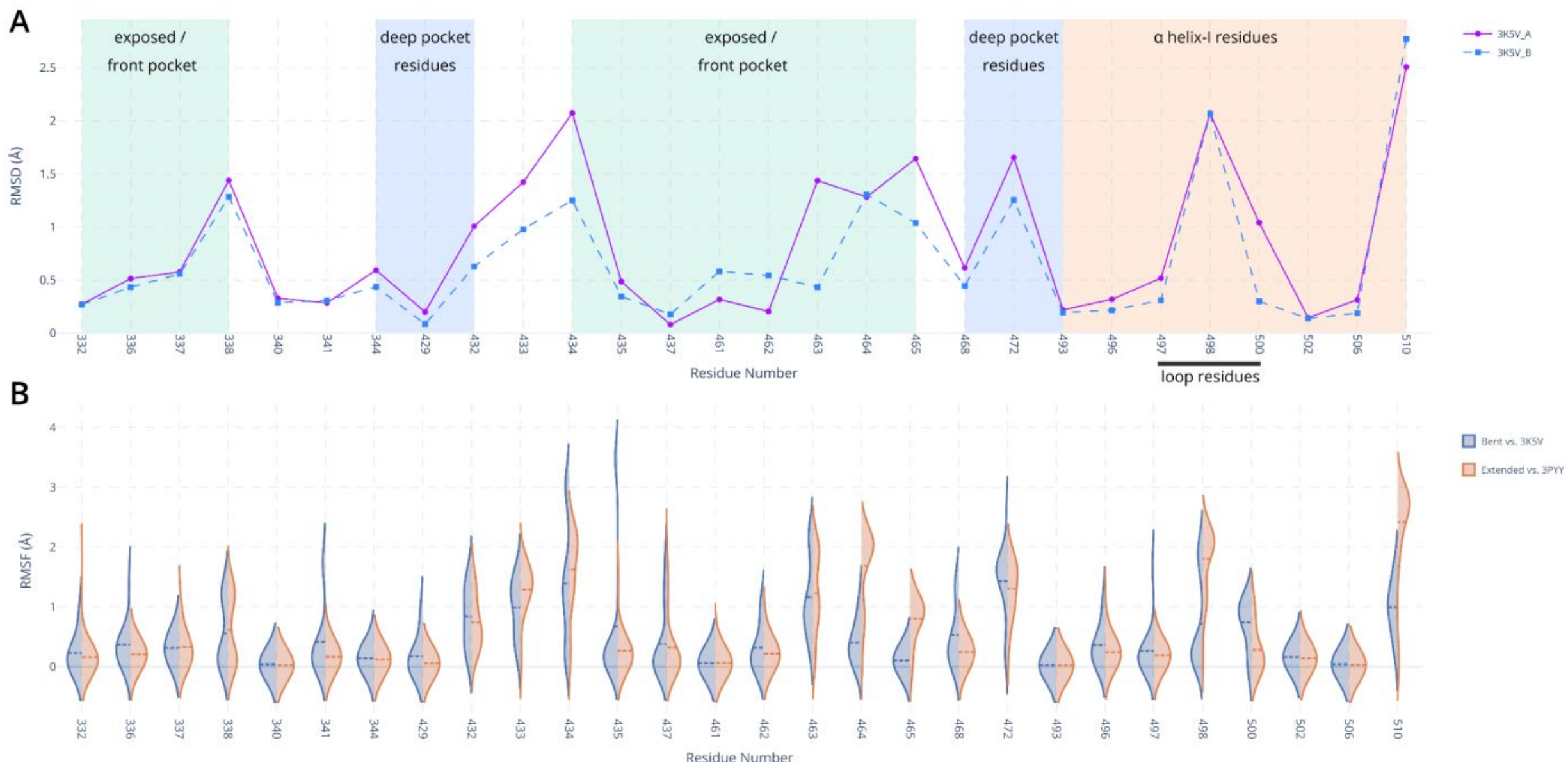


**Figure S2.** Abl crystallographic data analysis of structures with bound MP modulators (see **Table S1**). (A) All-atom RMSD for MP residues comparing 3PYY (chain B, extended α-helix-I) with 3K5V (bent α-helix-I). Residues are grouped into different pocket regions (exposed front pocket residues, deep pocket residues, and residues in the critical α-helix-I) and highlight how structures with different bound modulator classes differ in different parts of the pocket, not only in α-helix-I. (B) Per-residue RMSF after aligning individual residues on their backbone and $C_\beta$ to orient the side chain and highlight potential differing side chain conformations. All structures with a bent α-helix-I were compared against 3K5V chain A, and all structures with an extended α-helix-I were compared against 3PYY chain B. Structures labeled as 'cut' in **Table S1** were excluded from this analysis.

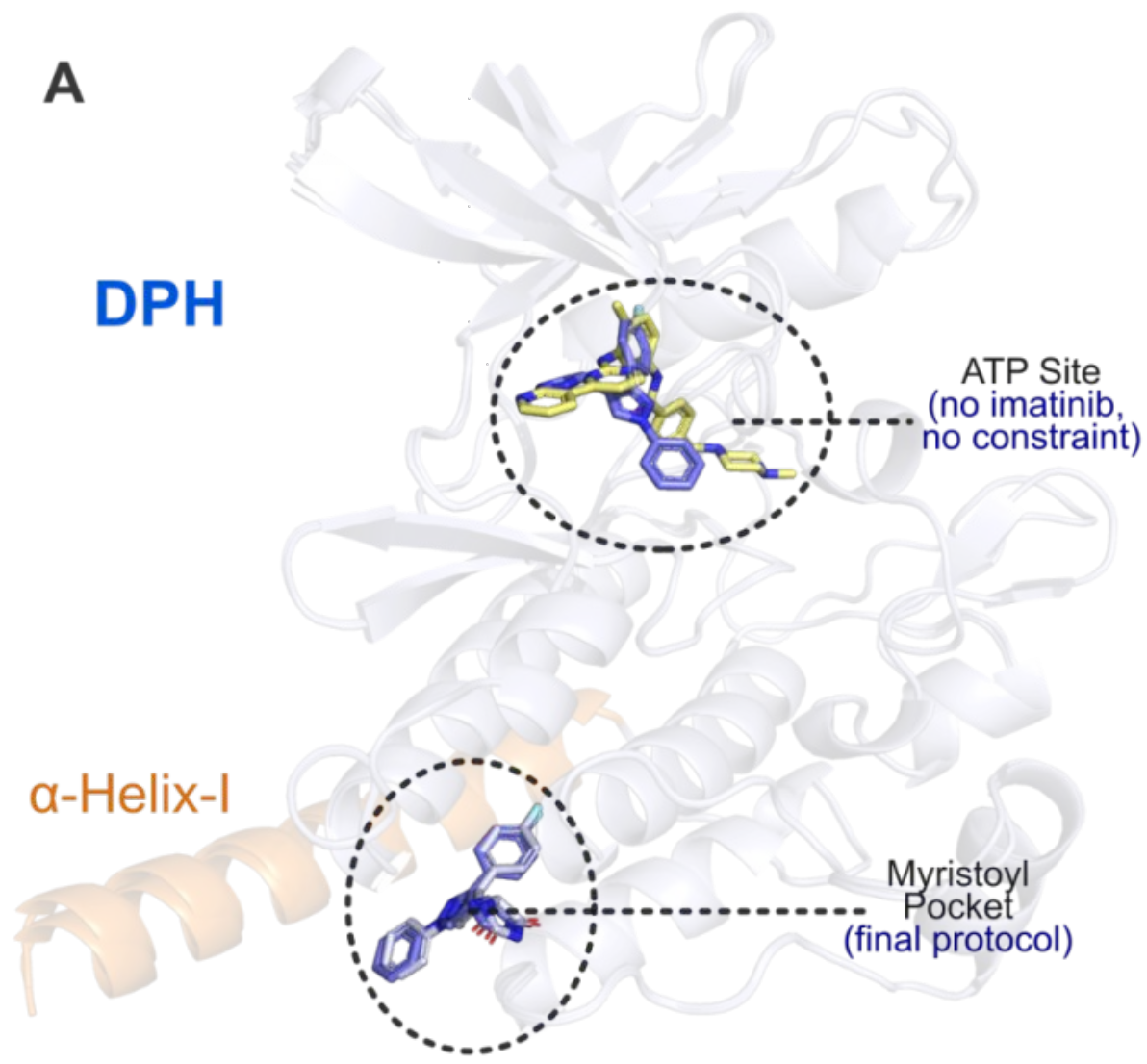


| Protocol | RMSD (Å) | Helix | Affinity Value | Affinity Prob. |
|---|---|---|---|---|
| Final | 0.539 | Extended | 0.580 | 0.215 |
| No imatinib, no constraint | 26.594 | Extended | 1.214 | 0.150 |

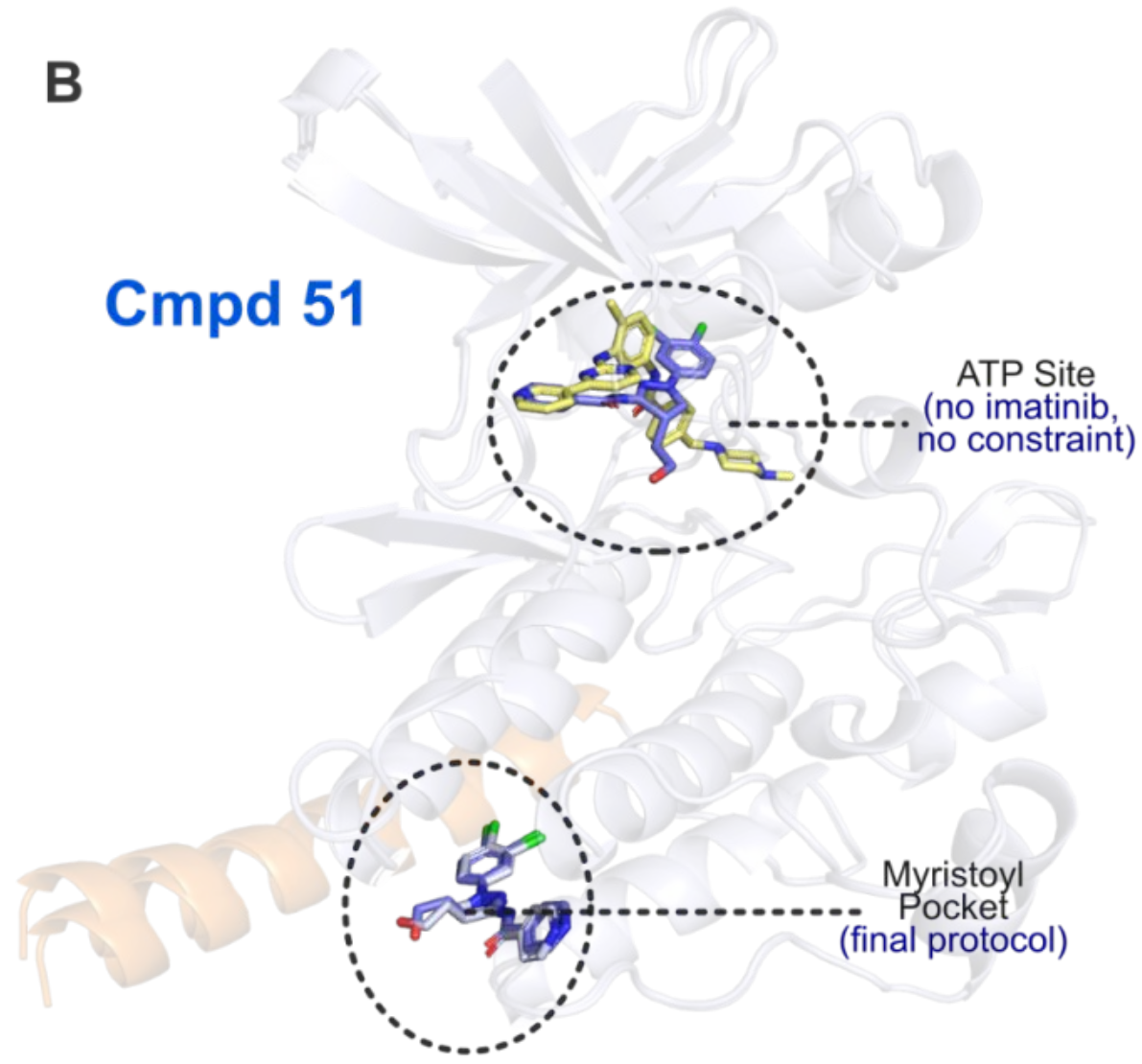


| Protocol | RMSD (Å) | Helix | Affinity Value | Affinity Prob. |
|---|---|---|---|---|
| Final | 0.461 | Extended | -0.903 | 0.790 |
| No imatinib, no constraint | 26.738 | Extended | -0.491 | 0.099 |

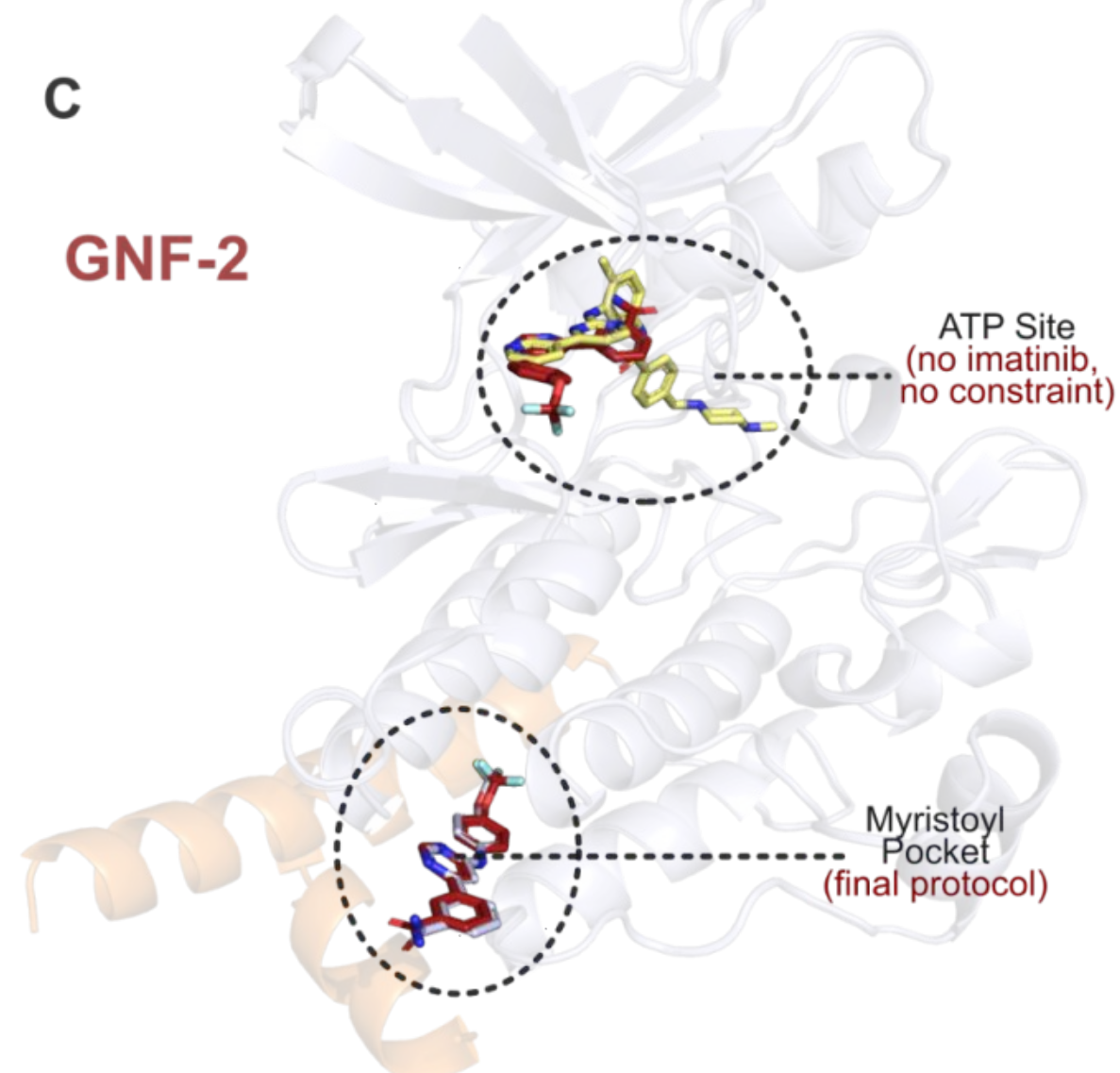


| Protocol | RMSD (Å) | Helix | Affinity Value | Affinity Prob. |
|---|---|---|---|---|
| Final | 0.502 | Bent | -1.195 | 0.791 |
| No imatinib, no constraint | 25.678 | Extended | 0.929 | 0.207 |

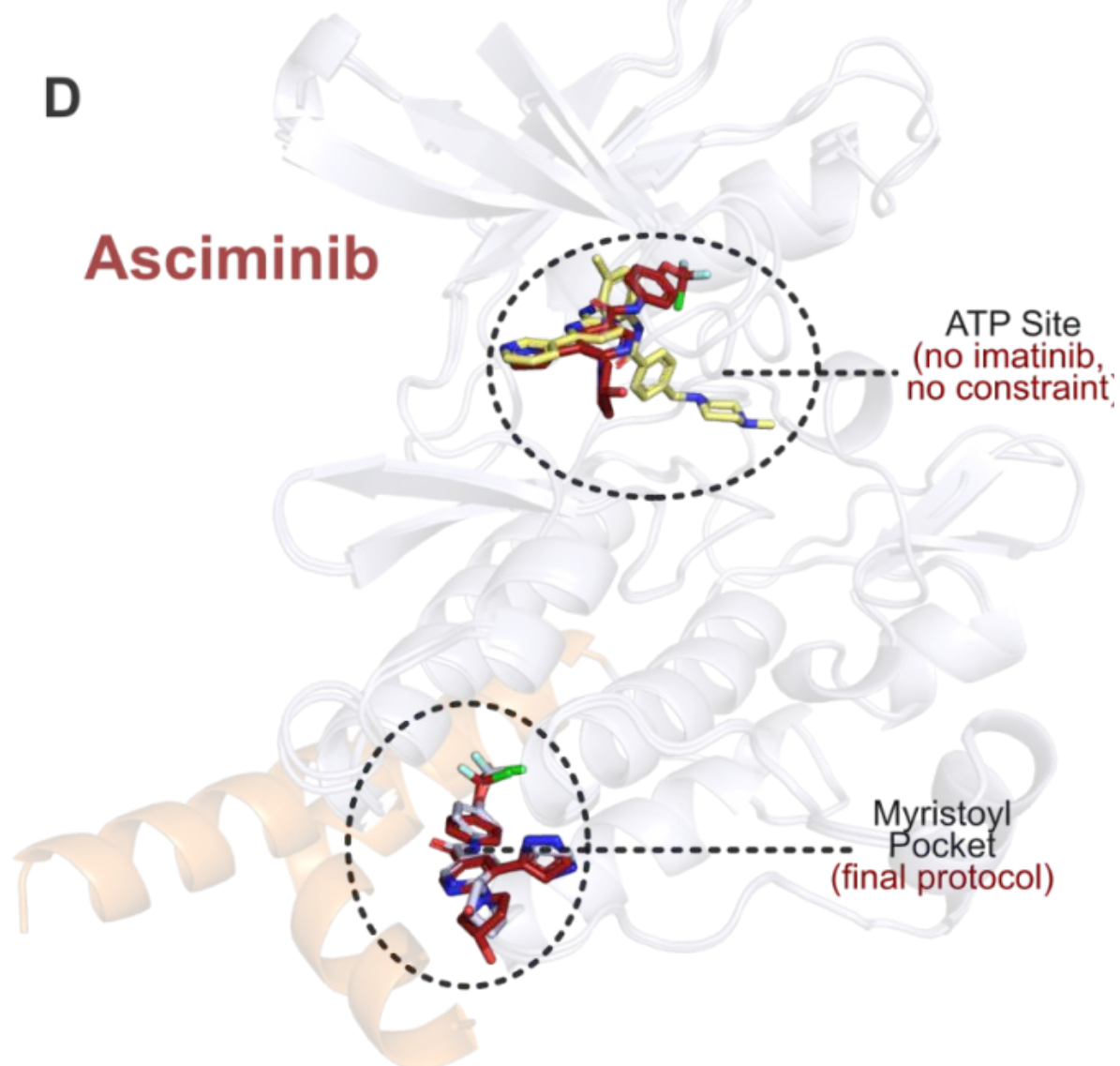


| Protocol | RMSD (Å) | Helix | Affinity Value | Affinity Prob. |
|---|---|---|---|---|
| Final | 0.817 | Bent | -2.034 | 0.863 |
| No imatinib, no constraint | 27.528 | Extended | -0.337 | 0.062 |

**Figure S3.** "Out of the box" co-folding without imatinib or constraints superimposed with the final protocol. (A) **DPH**; (B) **Cmpd51**; (C) **GNF-2**; (D) **Asciminib**. Activators are colored blue, inhibitors are colored red, their crystallographic structures are colored grey, and imatinib is colored yellow. Without imatinib or contact constraints on myristoyl pocket residues, the allosteric ligands were incorrectly directed to the ATP site, their affinity values and probabilities were lower, and the α-helix-I was inaccurately folded for the inhibitors.

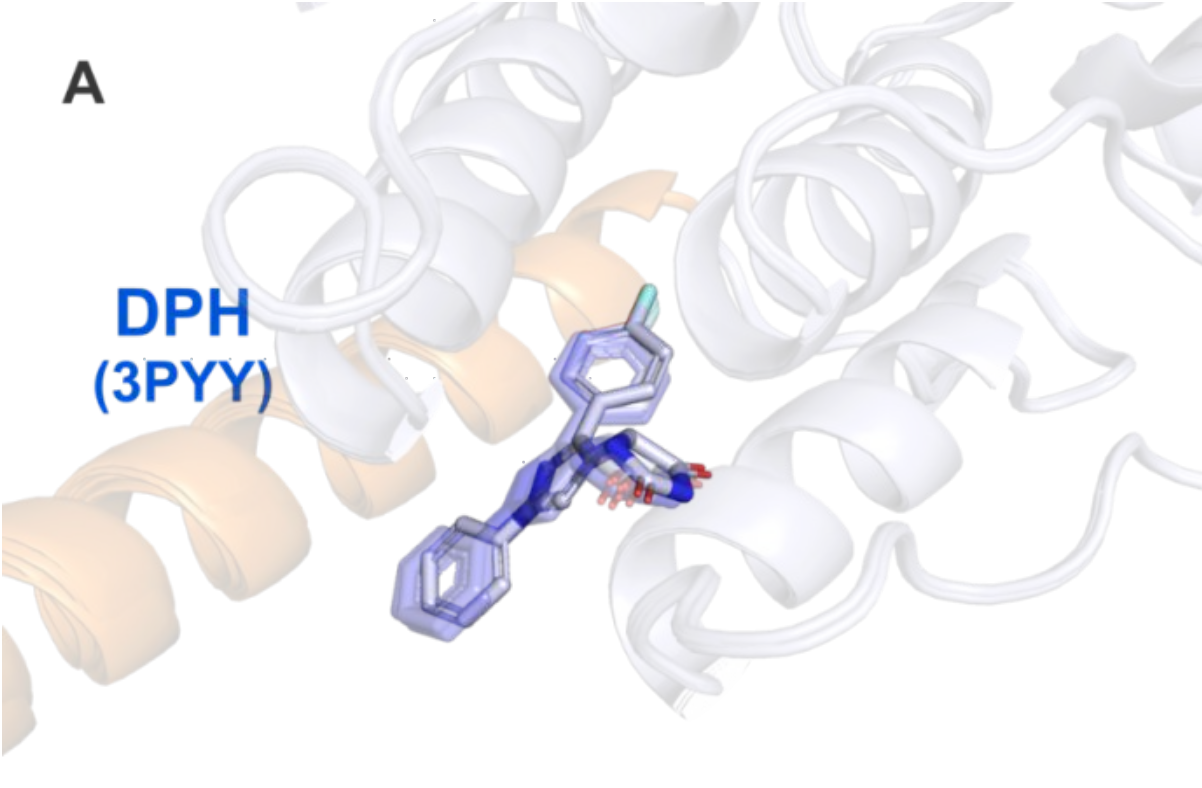


| Protocol | RMSD (Å) | Helix | Affinity Value | Affinity Prob. |
|---|---|---|---|---|
| P1 | 0.555 | Extended | 0.497 | 0.211 |
| P2 | 0.519 | Extended | 0.572 | 0.213 |
| P3 | 0.562 | Extended | 0.511 | 0.221 |
| P4 | 0.539 | Extended | 0.643 | 0.201 |
| P5 | 0.855 | Extended | 0.580 | 0.215 |

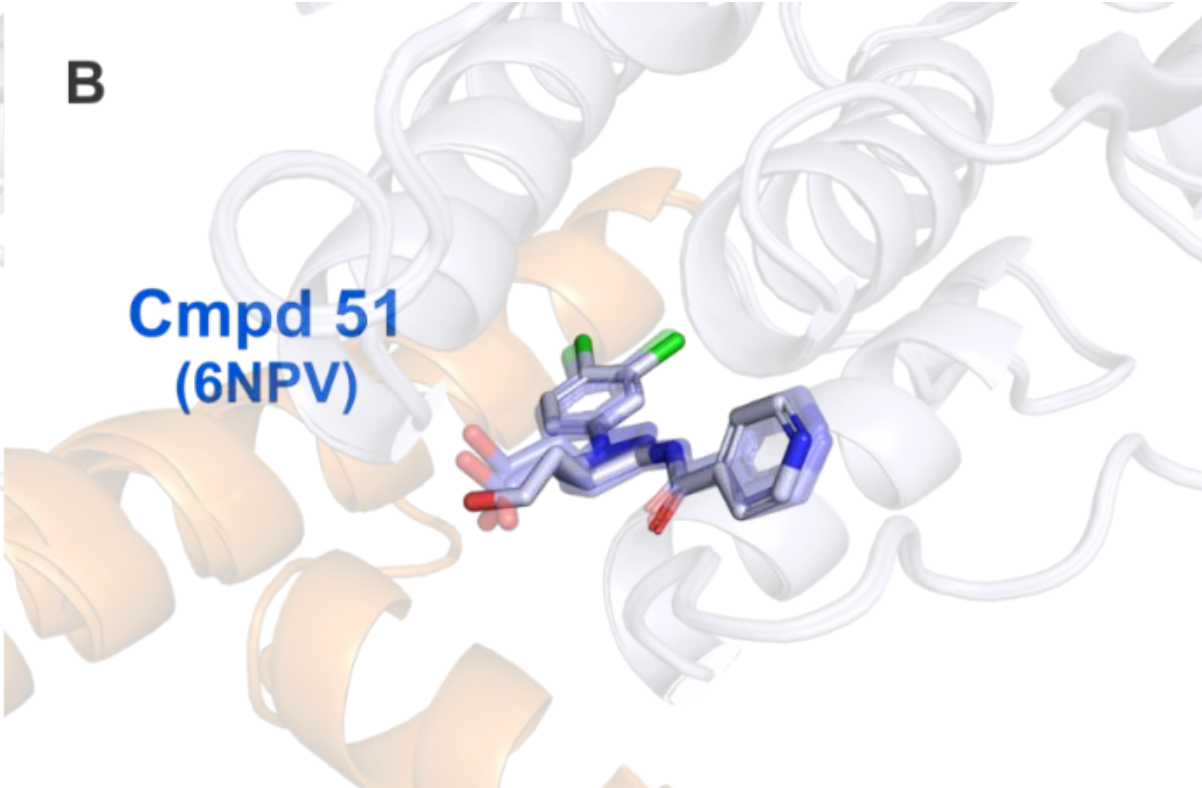


| Protocol | RMSD (Å) | Helix | Affinity Value | Affinity Prob. |
|---|---|---|---|---|
| P1 | 0.552 | Bent | -0.818 | 0.786 |
| P2 | 0.359 | Extended | -0.854 | 0.790 |
| P3 | 0.471 | Extended | -0.827 | 0.792 |
| P4 | 0.461 | Extended | -0.642 | 0.717 |
| P5 | 0.477 | Extended | -0.903 | 0.790 |

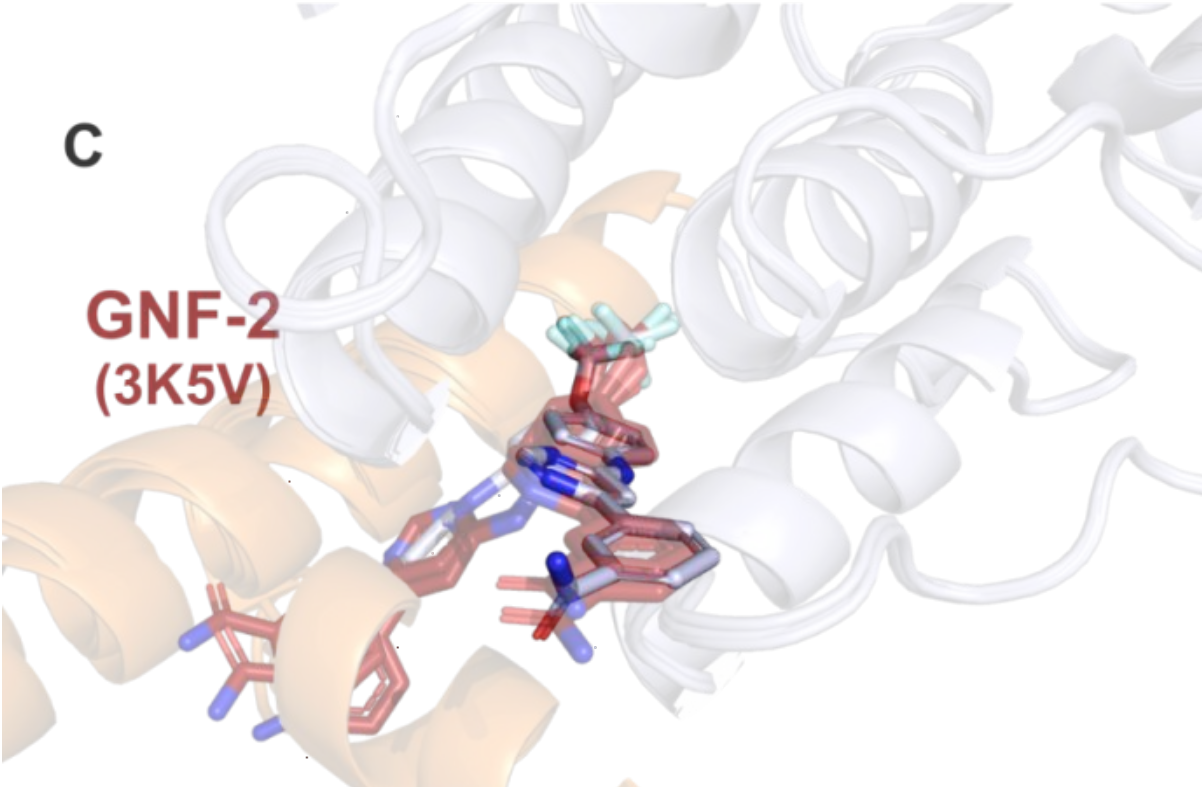


| Protocol | RMSD (Å) | Helix | Affinity Value | Affinity Prob. |
|---|---|---|---|---|
| P1 | 0.635 | Bent | -1.201 | 0.799 |
| P2 | 8.042 | Extended | -1.299 | 0.813 |
| P3 | 8.136 | Extended | -1.391 | 0.827 |
| P4 | 0.502 | Extended | -0.609 | 0.546 |
| P5 | 9.665 | Bent | -1.195 | 0.791 |

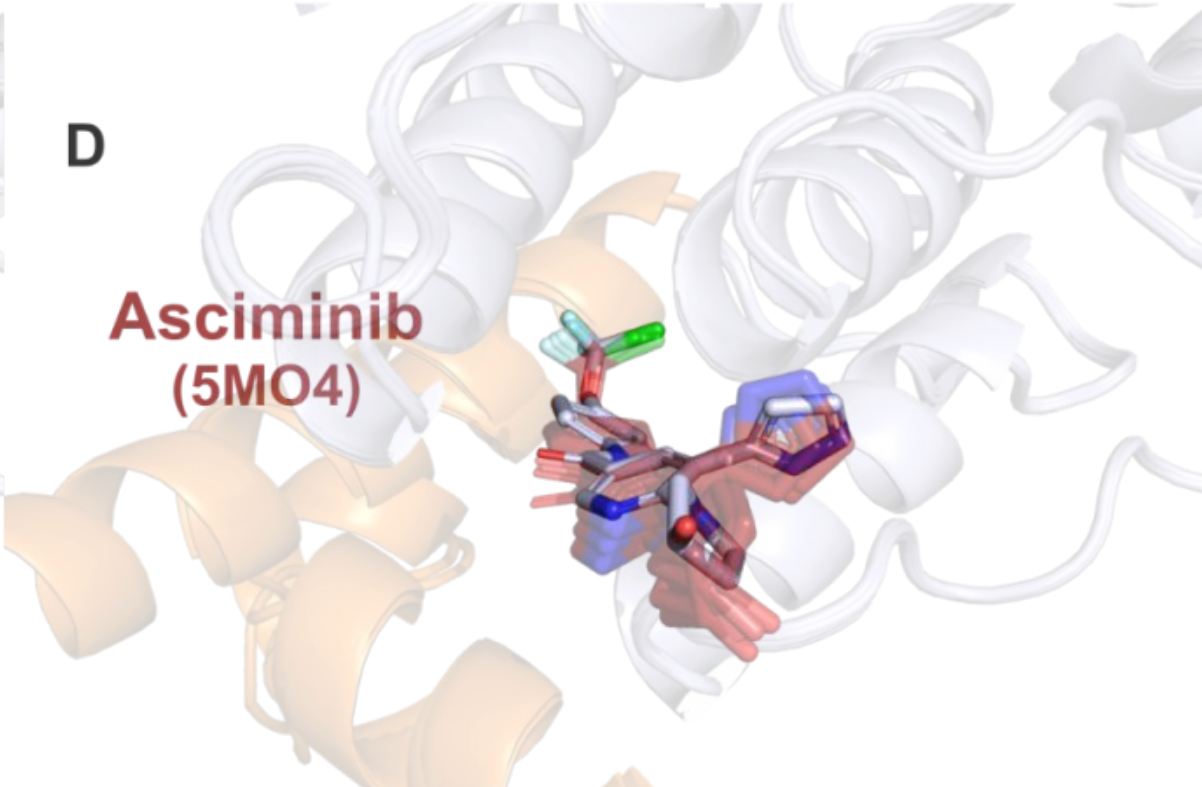


| Protocol | RMSD (Å) | Helix | Affinity Value | Affinity Prob. |
|---|---|---|---|---|
| P1 | 0.817 | Bent | -2.238 | 0.908 |
| P2 | 1.109 | Extended | -2.074 | 0.876 |
| P3 | 0.959 | Bent | -2.121 | 0.887 |
| P4 | 0.817 | Bent | -1.982 | 0.834 |
| P5 | 0.773 | Bent | -2.034 | 0.863 |

E

| Protocol | Template | Imatinib | Constraint |
|---|---|---|---|
| P1 | 3K5V | No | Yes |
| P2 | 3PYY | No | Yes |
| P3 | No | No | Yes |
| P4 | No | Yes | No |
| P5 | No | Yes | Yes |

**Figure S4.** Assessment of different co-folding protocols, superimposed with crystallographic structures. (A) **DPH**; (B) **Cmpd51**; (C) **GNF-2**; (D) **Asciminib**; (E) Protocol descriptions. P1 and P2 protocols used 3K5V and 3PYY as templates, respectively, while P3, P4 and P5 had no templates. P4 and P5 had imatinib in the ATP-binding pocket, and P4 was the only applied protocol without a contact constraint with residue Ala344 to direct the ligands to the MP. P5 was the only protocol to correctly predict helix bending for all ligands (extended for both activators and bent for both inhibitors), and was chosen for further analyses.

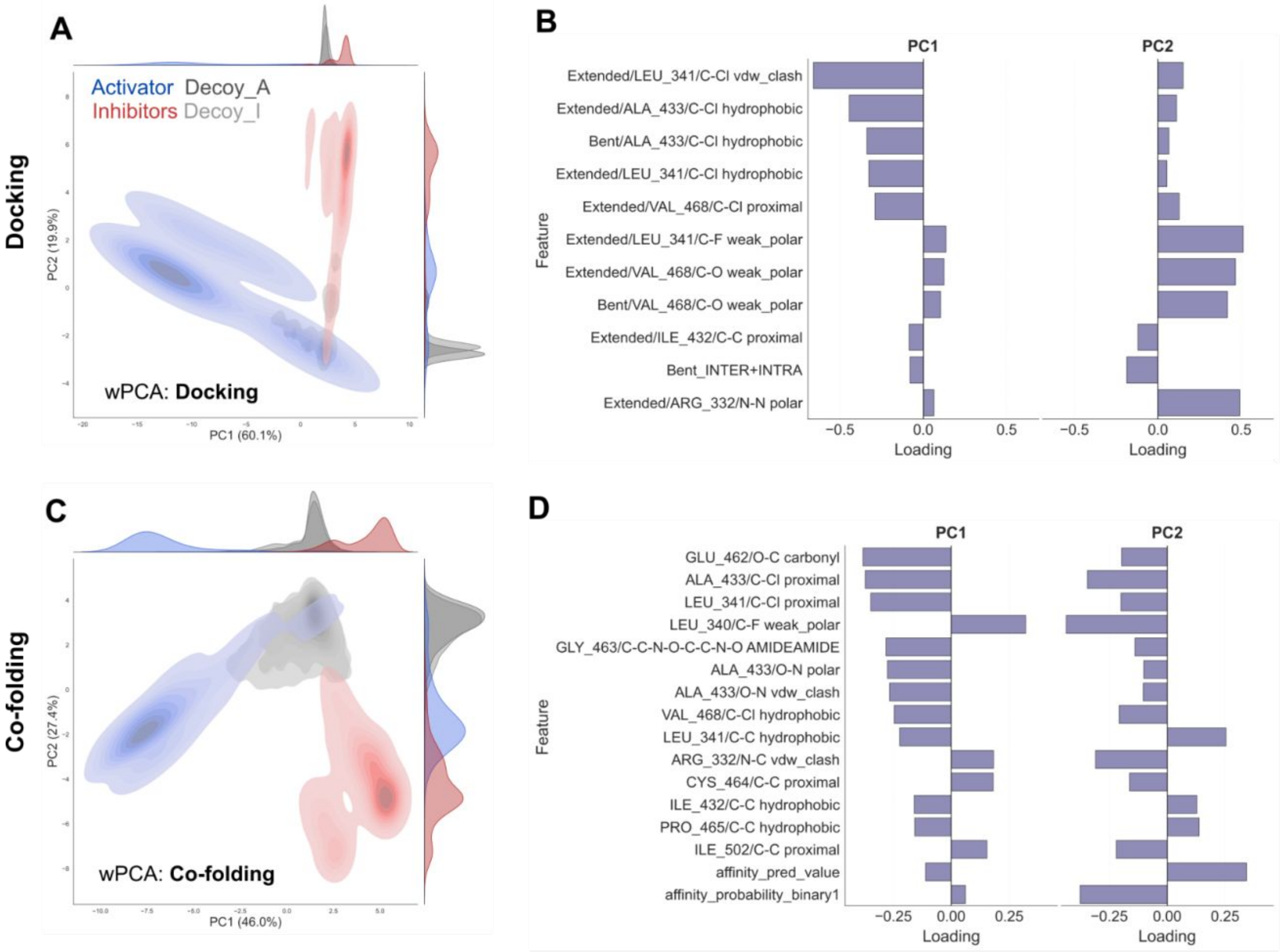


**Figure S5.** KDE plots of docking wPCA (A) and the PC loadings of its features (B); KDE plots of co-folding wPCA (C) and the PC loadings of its features (D).

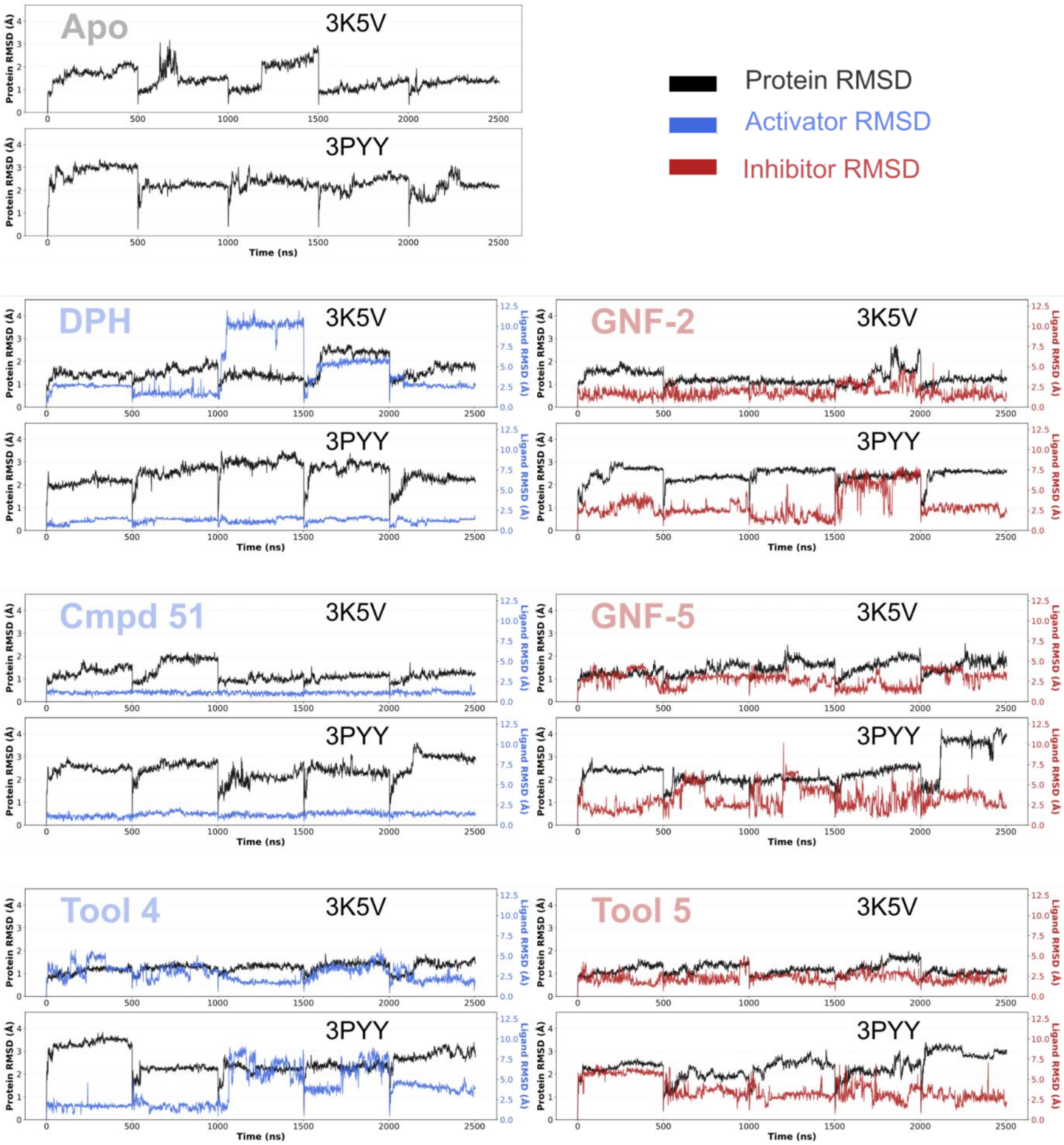


**Figure S6.** Root-mean square deviation of protein alpha carbons (black line) and ligand heavy atoms (blue line for activators, red line for inhibitors) in each trajectory, using the same scales. Simulation results were obtained from the merged trajectory of 5 replicas of 500 ns each.

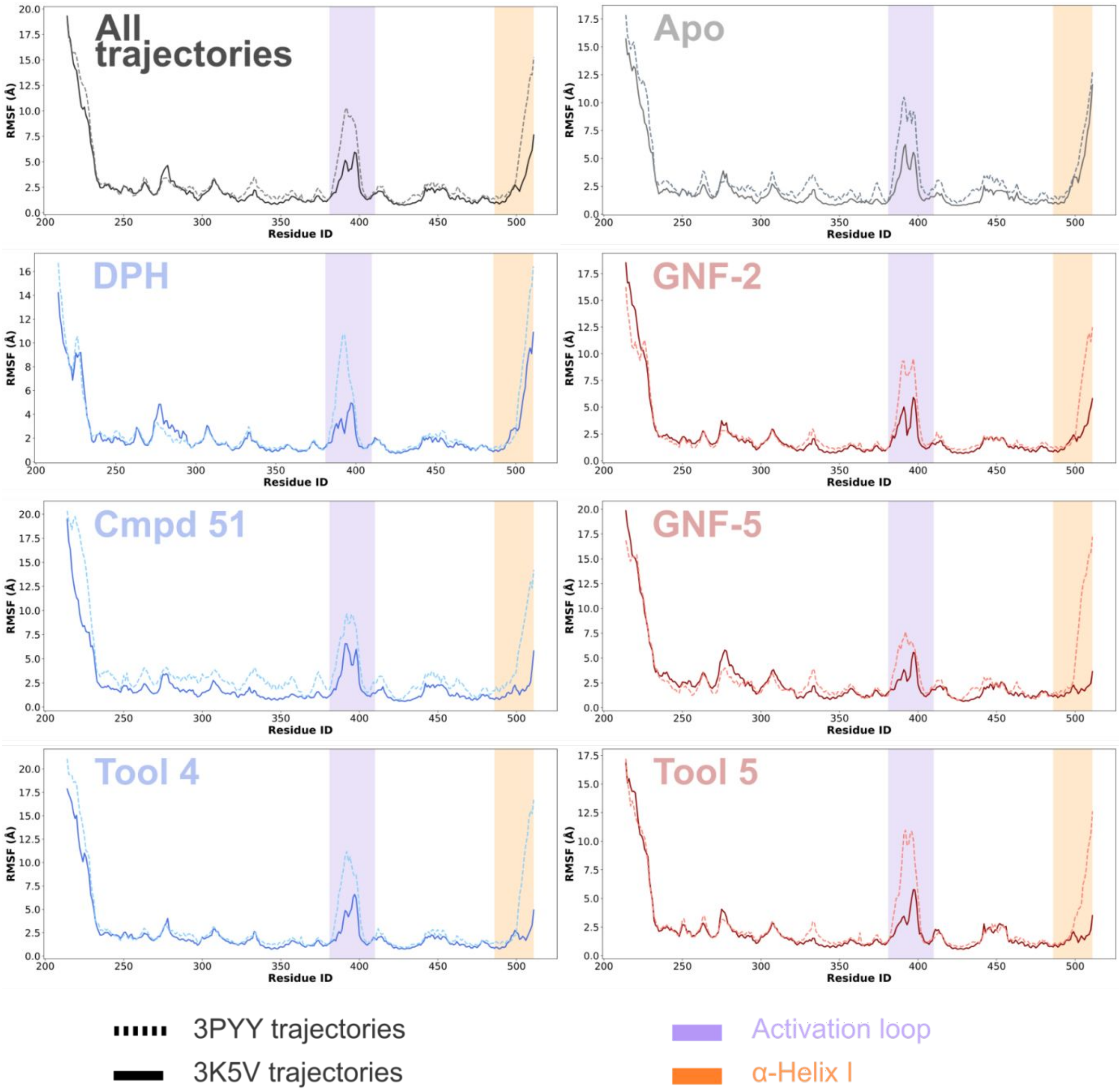


**Figure S7.** Root-mean square fluctuation (RMSF) of alpha carbons in combined trajectories and for each ligand individually. Dashed lines represent 3PYY model-based starting trajectories, and full lines represent 3K5V model-based starting trajectories. The activation loop region is colored purple, and the α-helix-I region is colored orange.

*Ligand Dynamics Within the MP Suggests Distinct Deep-Pocket Occupancy in MD Trajectories*

The distance between each ligand and the deep-pocket residues provides a proxy for ligand dynamics within the MP. This metric, defined as the distance between the ligand center of mass and the center of mass of deep-pocket residues Ala344, Leu429, Val468 and Phe493, was chosen to monitor ligand motion instead of ligand RMSD, due to the latter being biased by the solvent-exposed portion of the ligands farther away from the deep-pocket (**Figure S8**), which displays higher conformational flexibility and does not have an established SAR. **DPH**, an activator that has a steric clash with the helix in its bent conformation, exits the pocket when the 3K5V-based model (bent α-helix-I) was used to perform the MD simulation. Inhibitors generally exhibit longer occupancy times than activators (with the exception of **Cmpd51**), as illustrated by the time plots of **Tool 4** and **Tool 5** (**Figure S8**).

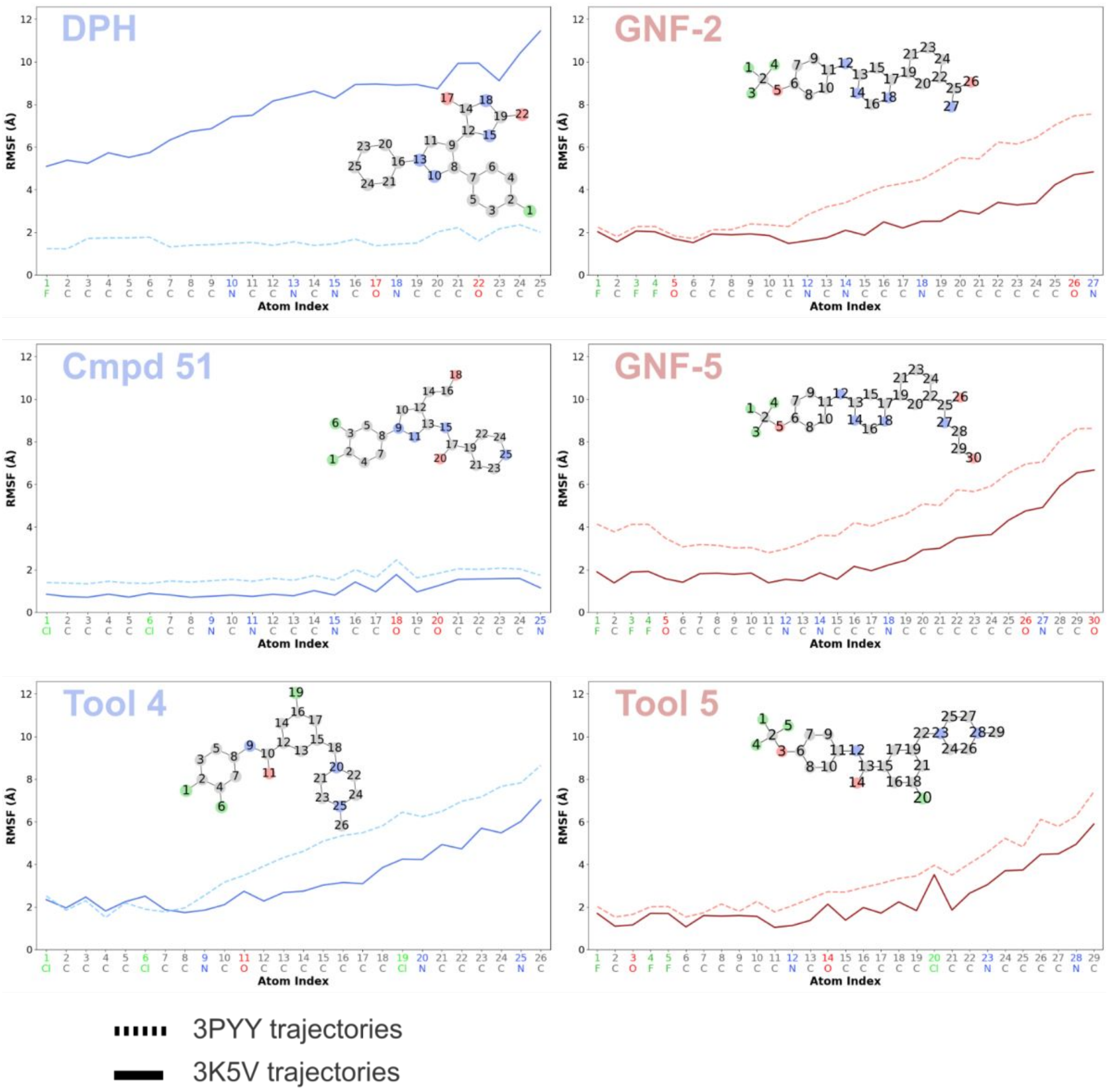


**Figure S8.** Heavy atom ligand root-mean square fluctuation (RMSF) for each ligand in their respective 3K5V and 3PYY model-based trajectories. For each ligand, atoms are numbered starting from closest to the deep-pocket (residues Ala344, Leu429, Val468, and Phe493 used as reference).

**Tool 4** (activator) and **Tool 5** (inhibitor) differ only in their substituents: a *meta*- and *para*-dichlorophenyl group in **Tool 4** versus a para-trifluoromethoxy group in **Tool 5**. Deng *et al.* (2010) explore the importance of the $-OCF_3$ group for inhibition, demonstrating that its substitution with fluoro or methoxy groups leads to loss of activity.[5] Removal of only the oxygen atom (converting from trifluoromethoxy to trifluoromethyl) was the only tolerated substitution for the group, suggesting that the $CF_3$ moiety is the key pharmacophoric motif. Consistent with this SAR, the inhibitor **Tool 5** maintains more stable deep-pocket occupancy, while the activator **Tool 4** exhibits greater positional fluctuation (**Figure S9F,G**). Although individually weak, numerous C-F interactions may help stabilize inhibitors within the deep pocket. Notably, interaction frequency analysis shows that the hydrophobic C-Cl interactions between **Tool 4** and deep-pocket residues (**Figure S9D**) occur more frequently in our MD simulations than the weak polar C-F interactions between **Tool 5** and the same residues (**Figure S9E**). This difference results from how the Arpeggio software computes each interaction type, using a distance threshold of 4.5 Å for hydrophobic interactions and 3.6 Å for weak polar interactions. We can hypothesize that, despite their higher frequency, C-Cl interactions remain stable at greater distances, leading to the higher deviations observed for **Tool 4** in comparison to **Tool 5** (**Figure S9A-C**).

Furthermore, the positioning of these substituents could influence stability: while one chlorine atom in **Tool 4** occupies the same *para* position as the $-OCF_3$ group, the other is in a *meta* position that could potentially introduce steric clashes within the deep pocket. However, **Cmpd51** shares the same meta- and para-chlorine substitution pattern (**Figure 1D**) yet exhibits the most stable MP residence among all ligands (**Figure 6A, Figure S9**). SAR from Simpson *et al.* (2019) shows that the dichlorophenyl group is optimal for activation in both enzyme and cellular assays, whereas a single Cl in either the *meta* or *para* position retains MP binding and enzyme activation but causes a lack of detectable cellular activation.[7]

In addition, Schoepfer *et al.* (2018), which discovered **Asciminib**, showed retention of inhibitory activity upon substitution of one fluorine atom in the $-OCF_3$ group with chlorine ($-OCF_2Cl$).[6] The authors report this was done after molecular modeling indicated that the larger chlorine could be accommodated deep within the pocket. Additionally, interaction frequency analysis revealed a stark contrast of hydrophobic contacts with residue Cys464 between **Tool 4** and **Tool 5** (**Figure 6C, D**), with 41.2% for **Tool 4** and 73.0% for **Tool 5**. This is consistent with the Boltz-2 wFW analysis, which highlighted this interaction as the most distinguishing feature between activators and inhibitors (**Figure 5B**).

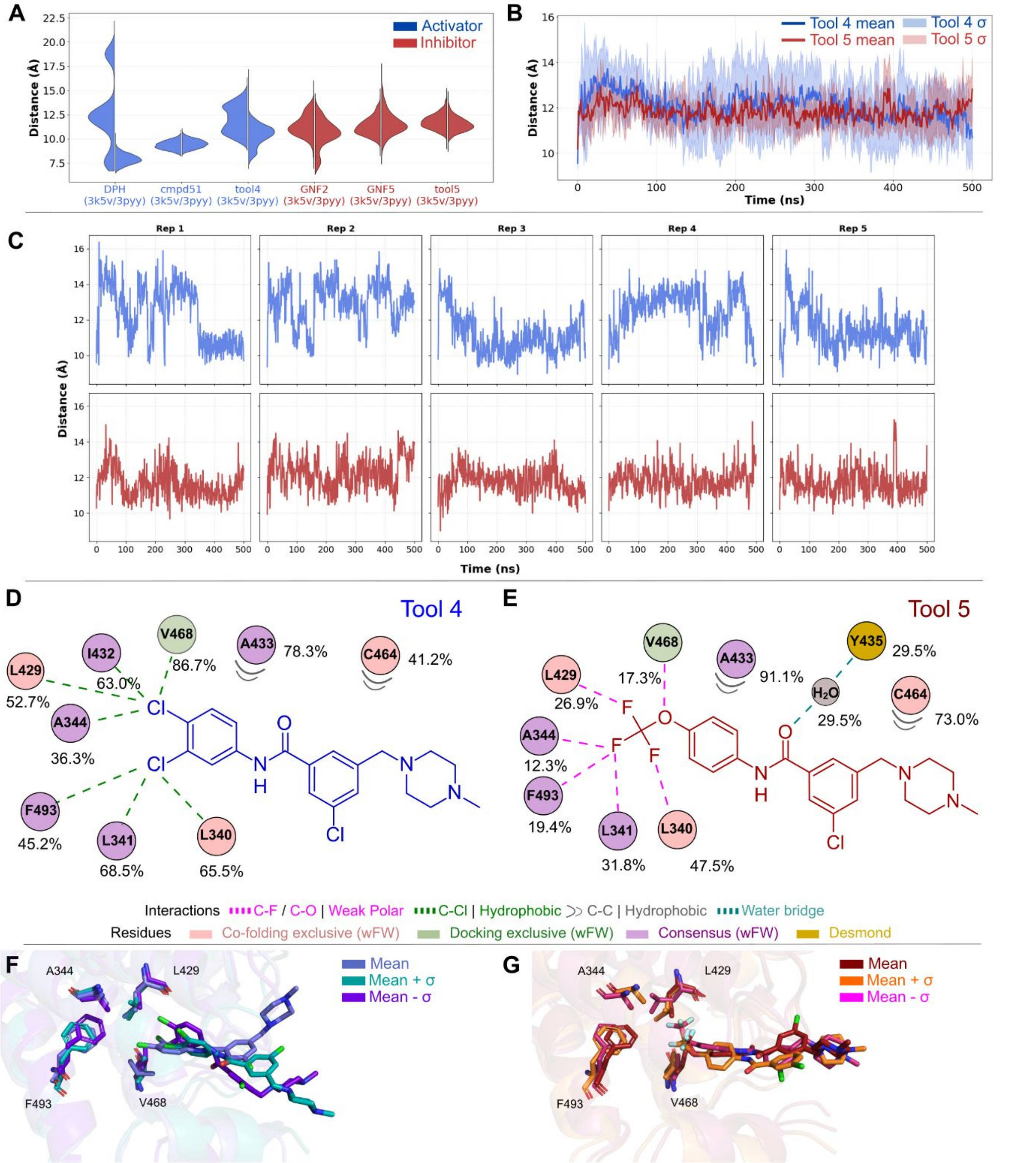


**Figure S9.** Ligand deep pocket stability in MD simulations. (A) Violin plot representing the distance distribution of ligand center of mass to deep pocket center of mass (residues Ala344, Leu429, Val468, and Phe493). (B) Mean ± standard deviation (σ) Ligand-MP distance over time of **Tool 4** (blue) and **Tool 5** (red). Both trajectory groups start from the 3K5V model (bent α-helix I conformation), with results obtained from the merged trajectory of 5 replicas, each 500 ns long. (C) Individual distance over time for each replica of **Tool 4** and **Tool 5** 3K5V trajectories. Diagrams showing the frequency of interactions (calculated using Arpeggio) in the top 20 wFW for the MD trajectories of **Tool 4** (D) and **Tool 5** (E). Structural representation of the ligand-deep pocket distances depicting a structure from the mean distance value, as well as representatives from distances' extremes (*i.e.*, mean ± standard deviation) for **Tool 4** (F) and **Tool 5** (G).

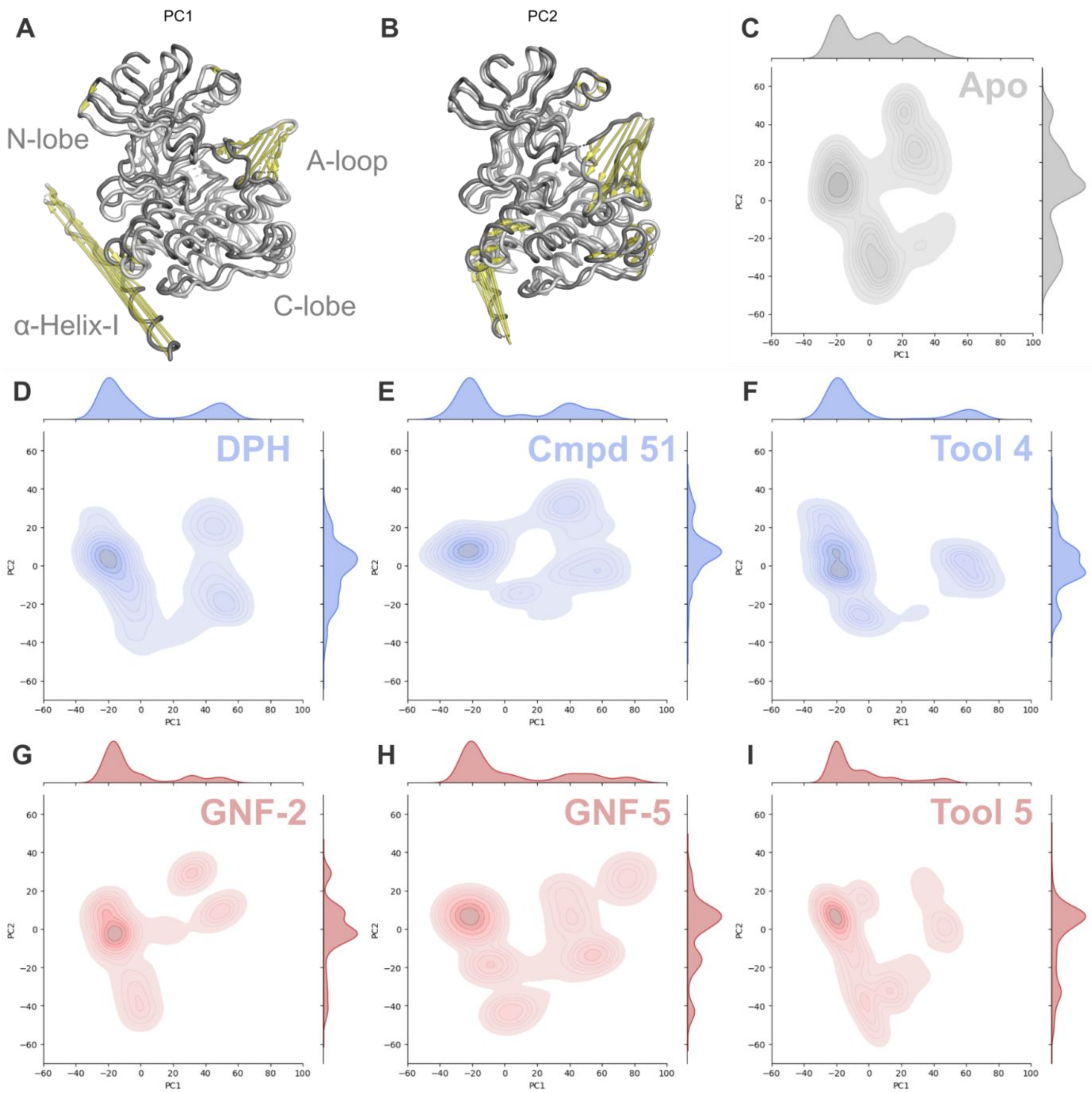


**Figure S10.** Global protein motions generated by the principal component analyses of all concatenated protein trajectories. (A) PC1 movement projections; (B) PC2 movement projections. KDE plots of all projected trajectories in $PCA_{all}$, colored by ligand class. Apo trajectories are colored gray, activator colored blue, and inhibitor colored red. (C) Apo trajectories; (D) **Cmpd51**; (E) **DPH**; (F) **Tool 4**; (G) **GNF-2**; (H) **GNF-5**; (I) **Tool 5**.

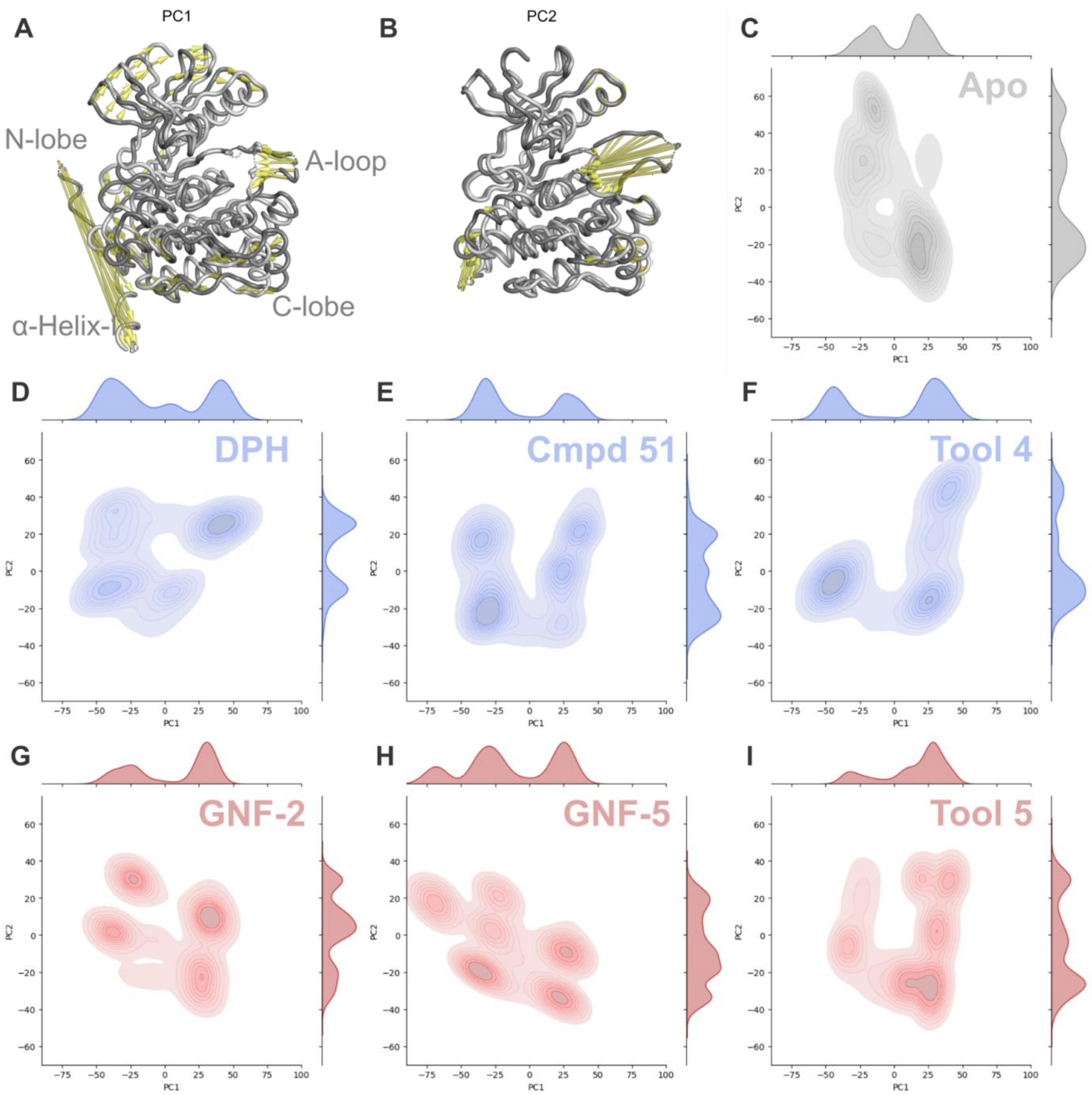


**Figure S11.** Global protein motions generated by the principal component analyses of all concatenated protein trajectories. (A) PC1 movement projections; (B) PC2 movement projections; KDE plots of all projected trajectories in $PCA_{3PYY}$, colored by ligand class. Apo trajectories are colored gray, activator colored blue, and inhibitor colored red. (C) Apo trajectories; (D) **Cmpd51**; (E) **DPH**; (F) **Tool 4**; (G) **GNF-2**; (H) **GNF-5**; (I) **Tool 5**.

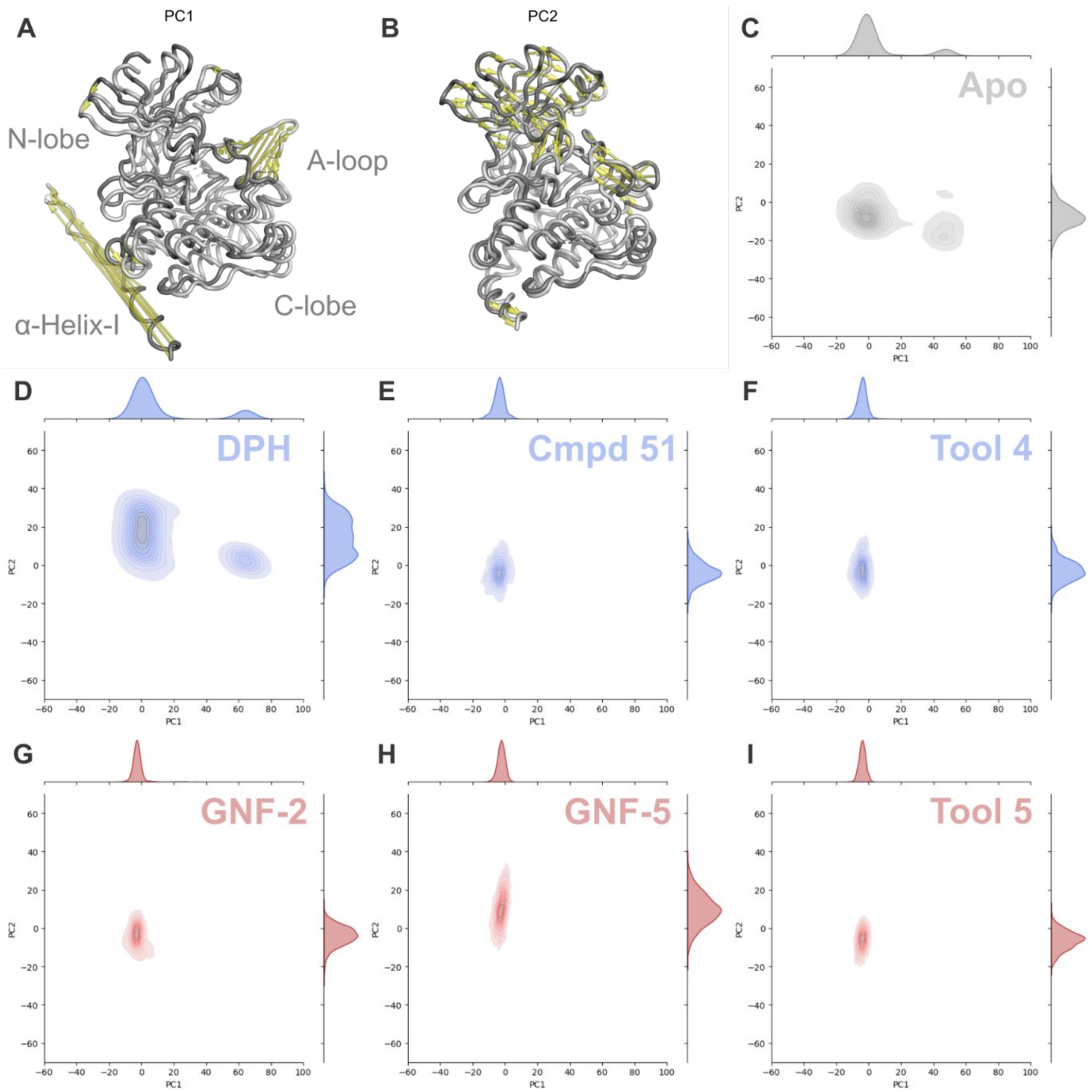


**Figure S12.** Global protein motions generated by the principal component analyses of all concatenated protein trajectories. (A) PC1 movement projections; (B) PC2 movement projections; KDE plots of all projected trajectories in $PCA_{3K5V}$, colored by ligand class. Apo trajectories are colored gray, activator colored blue, and inhibitor colored red. (C) Apo trajectories; (D) **Cmpd51**; (E) **DPH**; (F) **Tool 4**; (G) **GNF-2**; (H) **GNF-5**; (I) **Tool 5**.
